# PDGFRα demarcates the cardiogenic clonogenic Sca1^+^ stem/progenitor cell in adult murine myocardium

**DOI:** 10.1038/ncomms7930

**Published:** 2015-05-18

**Authors:** Michela Noseda, Mutsuo Harada, Sara McSweeney, Thomas Leja, Elisa Belian, Daniel J. Stuckey, Marta S. Abreu Paiva, Josef Habib, Iain Macaulay, Adam J. de Smith, Farah al-Beidh, Robert Sampson, R. Thomas Lumbers, Pulivarthi Rao, Sian E. Harding, Alexandra I. F. Blakemore, Sten Eirik Jacobsen, Mauricio Barahona, Michael D. Schneider

**Affiliations:** 1 British Heart Foundation Centre of Research Excellence, Imperial College London, London SW7 2AZ, UK; 2 National Heart and Lung Institute, Imperial College London, London SW7 2AZ, UK; 3 Centre for Advanced Biomedical Imaging (CABI), University College London, London WC1E 6DD, UK; 4 Department of Biomedical Engineering, King's College London, London SE1 7EH, UK; 5 Haematopoietic Stem Cell Biology, MRC Weatherall Institute of Molecular Medicine, University of Oxford, Oxford OX3 9DS, UK; 6 Department of Medicine, Imperial College London, London SW7 2AZ, UK; 7 Department of Epidemiology and Biostatistics, University of California, San Francisco, San Francisco, California 94143, USA; 8 Texas Children's Cancer Center, Baylor College of Medicine, Houston, Texas 77030, USA; 9 Department of Pediatrics, Baylor College of Medicine, Houston, Texas 77030, USA; 10 Department of Mathematics, Imperial College London, London SW7 2AZ, UK

## Abstract

Cardiac progenitor/stem cells in adult hearts represent an attractive therapeutic target for heart regeneration, though (inter)-relationships among reported cells remain obscure. Using single-cell qRT–PCR and clonal analyses, here we define four subpopulations of cardiac progenitor/stem cells in adult mouse myocardium all sharing stem cell antigen-1 (Sca1), based on side population (SP) phenotype, PECAM-1 (CD31) and platelet-derived growth factor receptor-α (PDGFRα) expression. SP status predicts clonogenicity and cardiogenic gene expression (*Gata4/6*, *Hand2* and *Tbx5/20*), properties segregating more specifically to PDGFRα^+^ cells. Clonal progeny of single Sca1^+^ SP cells show cardiomyocyte, endothelial and smooth muscle lineage potential after cardiac grafting, augmenting cardiac function although durable engraftment is rare. PDGFRα^−^ cells are characterized by *Kdr/Flk1*, *Cdh5*, CD31 and lack of clonogenicity. PDGFRα^+^/CD31^−^ cells derive from cells formerly expressing *Mesp1*, *Nkx2-5*, *Isl1*, *Gata5* and *Wt1*, distinct from PDGFRα^−^/CD31^+^ cells (*Gata5* low; *Flk1* and *Tie2* high). Thus, PDGFRα demarcates the clonogenic cardiogenic Sca1^+^ stem/progenitor cell.

## Abstract (web summary)

Adult cardiac progenitor/stem cells (CPSCs) possess valuable potential for heart repair that is limited by the elusiveness of these cells. Here Noseda *et al.* refine the definition of murine CPSCs producing stem cell antigen 1 (Sca1), mapping the cardiogenic signature and clonogenicity to the subgroup of Sca1^+^ cells expressing PDGFRα.

## Graphical abstract

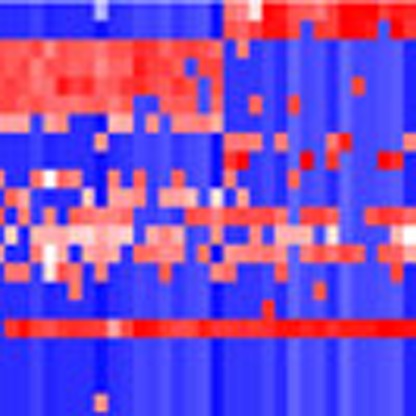

Fate-mapping studies provide evidence that adult mammalian cardiac regeneration exists, though at a level insufficient to rescue damaged hearts, occurring at least partly through a lineage decision by progenitor/stem cells^1^,^2^,^3^,^4^, not proliferation of pre-formed myocytes as in zebrafish or newborn mice^5^,^6^. This view is supported by evidence using transgenic fluorescent anillin, that cardiomyocytes in damaged adult hearts increase in ploidy but do not divide^7^. Characterizing the dormant adult cardiac progenitors is arguably still in its infancy, despite identifiers including the orphan receptor stem cell antigen-1 (Sca1; refs 2, 3, 8, 9), c-kit^4^,^10^, side population (SP) dye-efflux phenotype^11^,^12^,^13^, *Isl1* (ref. 14), cardiosphere-15 and colony-forming assays^16^, aldehyde dehydrogenase^17^, or re-expression of the embryonic epicardial marker *Wt1* (ref. 18). Notwithstanding these uncertainties, cardiac progenitor/stem cells have begun to be used in human trials^19^. Unlike cells from bone marrow, intrinsic progenitor/stem cells residing in the heart are predisposed to convert to the cardiac muscle lineage after grafting^5^ and are, uniquely, a possible target for activation *in situ* by developmental catalysts^5^,^18^.

Existing work on endogenous cardiac progenitor cells has chiefly relied on purified but potentially mixed populations. Where clonal growth was reported, this was often achieved at a prevalence ≤0.1% for fresh cells, or contingent on prior adaptation to culture^10^,^20^,^21^,^22^,^23^,^24^. In one study, only 0.03% of adult cardiac Sca1^+^ cells proliferated beyond 14 days^20^. Sheets of clonally expanded Sca1^+^ cells improve cardiac function after infarction^21^. Sca1^+^ cells have cardiogenic and vascular differentiation potential^2^,^8^,^9^,^12^, though whether their single-cell progeny have multilineage potential is uncertain.

Tracking cell progeny with Cre recombinase suggests that Sca1-fated cells generate cardiac muscle *in situ* during normal ageing^3^ and that Sca1^+^ cells are a major source of new myocytes after ischaemic injury^2^. Fate mapping with *R26R-Confetti*, which labels clonal derivatives randomly with one of four fluorescent proteins^25^, suggests that most single Sca1 cells have limited expansion potential and give rise to a single cell type^3^. Given this rare clonogenic potential, infrequent differentiation and variable expression of other markers^8^,^9^,^12^,^26^, Sca1^+^ cells, if not further refined, are presumptively heterogeneous. More remains to be learned about the native cells' capacity for clonal expansion and differentiation, including what prospective markers best predict the dual features of clonal growth plus enrichment for cardiogenic genes.

One candidate is the SP dye-efflux phenotype, which distinguishes hematopoietic stem cells with long-term self-renewal potential^27^ and is mediated by ATP-binding cassette (ABC) transport proteins associated with ‘stemness.' SP cells are found in developing and adult hearts^8^,^11^ and have greater colony-forming activity in methylcellulose than non-SP cells^12^, yet little information exists for cardiac SP single-cell derivatives. A predictor of the cardiac colony-forming units (cCFU-F) amid adult Sca1^+^ cells is platelet-derived growth factor receptor-α (PDGFRα); these multipotent cells resemble other mesenchymal stem cells (MSCs) and originate from the epicardium^16^. Relative to MSCs from other tissues, cCFU-Fs express greater *Mef2c*, but lack most cardiogenic transcription factors^26^.

In short, there is insufficient detailed information about the molecular characteristics of progenitor/stem cells residing in the adult heart, whether cloned cardiac cells faithfully resemble their *in situ* precursors and whether they resemble the multipotent cardiovascular progenitors in embryos and differentiating embryonic stem cells (ESCs). Despite the need to define more clearly the putative reservoirs of adult cardiac cells with differentiation potential, too little is known about how the various reported progenitors relate to one another. In particular, can one identify a more homogenous population at the single-cell level?

Here we have dissected the cardiac Sca1^+^ cells—based on their SP phenotype, PECAM-1 (CD31) and PDGFRα—using single-cell expression profiles and rigorous clonal analysis. SP status predicted clonogenicity plus the cardiogenic signature. However, both properties map even more selectively to PDGFRα^+^ cells.

## Results

### A cardiogenic signature in SP cells by single-cell profiling

**Figure 1:**
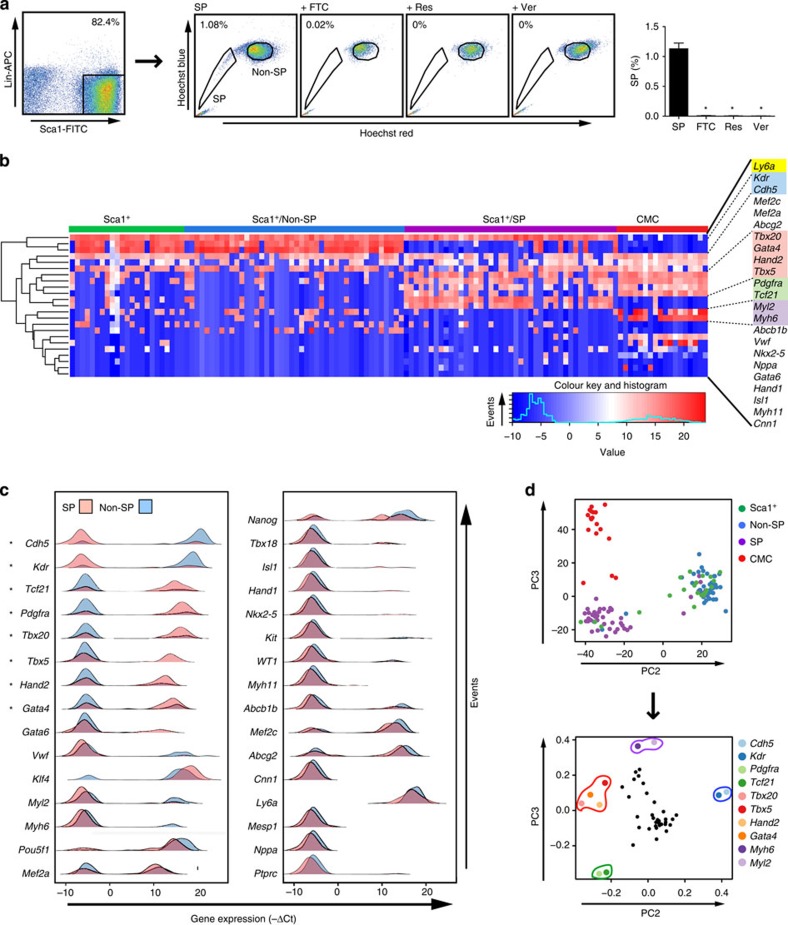
Single-cell profiles map cardiogenic gene expression to Sca1^+^ SP cells co-expressing *Pdgfra.* (**a**) Left, flow sorting of fresh cardiac Lin^−^ Sca1^+^ cells after immunomagnetic enrichment for Sca1. Center, further purification of Lin^−^ Sca1^+^ cells for the SP phenotype by Hoechst 33324 staining±ABC transporter inhibitors: FTC, fumitremorgin C; Res, reserpine; Ver, verapamil. Right, bar graph, mean±s.e.m.; *n*=4; *P≤0.0001. (**b**) Single-cell qRT–PCR profiles of fresh total Sca1^+^, SP and non-SP cells, compared with cardiomyocytes (CMC). The heatmap illustrates expression as −ΔCt values (blue, low or absent; red, high) and hierarchical clustering reveals the co-expression of functionally related genes in the populations indicated. Highlighted are: yellow, the Sca1 gene *Ly6a*; blue, *Kdr* and *Cdh5*, enriched in Sca1^+^ and non-SP cells; red, cardiogenic transcription factors, enriched in SP cells and CMC; green, *Pdgfra* and *Tcf21*, enriched in SP cells; violet, CMC genes. Sca1^+^, *n*=23; non-SP, *n*=44; SP, *n*=43; CMC, *n*=18. For the full set of 44 genes, see Supplementary Fig. 1. (**c**) Density plots of expression (−ΔCt) for selected genes in SP (light red) versus non-SP (light blue) cell populations. Genes are ordered according to increasing *P*-values. Those with a significantly divergent prevalence of expression between SP and non-SP cells are indicated by an asterisk. (**d**) PCA of the single-cell expression profiles. (Top) PC2 (19% variability) separates SP from non-SP sample scores, whereas PC3 (9% variability) establishes a distinct separation between SP/non-SP/Sca1^+^ cells and CMC. Non-SP and unfractionated Sca1^+^ cells cluster together in the PC projection. (Bottom) Gene loadings contributing to each PC indicate that a small subset of genes explain the cross-group variability captured by PC2 and PC3. *Cdh5* and *Kdr* are predominantly associated with non-SP and unfractionated Sca1^+^ cells, while *Pdgfra* and *Tcf21* are correlated with SP cells (as given by PC2). Differences between CMCs and the remaining samples are strongly reflected in PC3, with cardiac structural genes (*Myh6* and *Myl2*) clustered consistently.

To address the innate heterogeneity of the cardiac Sca1^+^ population, single-cell qRT–PCR (PCR with quantitative reverse transcription) was performed on fresh cells, obviating potential bias from *in vitro* expansion. Given that adult cardiac Sca1^+^ cells are enriched for SP cells with cardiogenic potential *in vivo*^8^,^11^,^12^,^13^, we tested whether SP cells possess a distinguishable profile. Adult cardiac Lin^−^/Sca1^+^ cells were isolated by immunomagnetic purification, further purified by flow cytometry (Fig. 1a, left), and stained with Hoechst 33342 (Fig. 1a, centre, right). SP cells comprised roughly 1% of the Lin^−^/Sca1^+^ population. The SP phenotype was suppressed by reserpine and verapamil, broad-spectrum inhibitors of ABC transport proteins, and by fumitremorgin C, more selective for ABCG2^27^. Gates for preparative sorting were widely spaced and non-contiguous, to obviate crossover by the ‘tails' of SP and non-SP cells.

Single Lin^−^ Sca1^+^ cells from the total, SP and non-SP fractions, plus single neonatal cardiomyocytes, were assayed by qRT–PCR (Fig. 1b–d; Supplementary Fig. 1). The Sca1 gene *Ly6a* was expressed in all Sca1^+^, SP and non-SP cells, as predicted from their purification via Sca1 (Fig. 1b,c). *Ly6a* was not expressed in myocytes, which had near-uniform expression of sarcomeric genes (*Myh6* and *Myl2*) and most cardiogenic transcription factors (*Gata4*, *Hand2*, *Mef2a/c*, *Nkx2-5* and *Tbx5/20*); *Gata6* was more rarely detected.

Among unfractionated Sca1^+^ cells, two complementary patterns of expression were resolved: a major population (87%) expressing vascular endothelial cadherin (*Cdh5*) and vascular endothelial growth factor receptor 2 (*Kdr*), and a minor population (13%) expressing genes for the mesodermal marker *Pdgfra* and *Tcf21*, a transcription factor that governs epicardial progenitor cell fate^28^,^29^. Typically, non-SP cells expressed *Cdh5* and *Kdr*, with *Mef2c* and *Mef2a* the only prevalent cardiac transcription factors (>90% *Cdh5*^+^/*Kdr*^+^; >75% *Mef2c*^*+*^). Hence, non-SP cells (*Pdgfra*^−^/*Tcf21*^−^/*Cdh5*^+^/*Kdr*^+^ and lacking other cardiogenic markers) resembled and likely account for the major Sca1^+^ population.

The few unfractionated Sca1^+^ cells lacking *Cdh5* and *Kdr* expression were enriched instead for *Pdgfra* and *Tcf21* and cardiac transcription factors (*Gata4*, *Hand2* and *Tbx5/20*). These cells did not express cardiomyocyte markers, excluding myocyte contamination as the source of this cardiogenic signature. The *Pdgfra*^+^/*Tcf21*^+^/*Cdh5*^−^/*Kdr*^−^ phenotype was rare in non-SP cells (11%) but predominated among SP cells (86%; Fig. 1b,c). *Gata4*, *Gata6*, *Tbx5*, *Tbx20* and *Hand2* were most prevalent, with little or no expression of *Hand1*, *Isl1* and *Nkx2-5*, or markers of cardiomyocytes (*Myl2* and *Myh6*), smooth muscle (SM; *Cnn1* and *Myh11*) and endothelium (*Vwf*). Thus, SP cells resemble the minor sub-population of unfractionated Sca1^+^ cells. The three Sca1^+^ populations expressed little or no *Kit* (Fig. 1c; Supplementary Fig. 1), which may signify a coexisting cell^4^,^10^ or precursor–product relationship.

By principal component analysis (PCA; Fig. 1d and Supplementary Fig. 2), SP cells, non-SP cells and cardiomyocytes were resolved as discrete groups, with the mixed Sca1^+^ population straddling its SP and non-SP fractions (Fig. 1d, upper panel). This separation of SP cells, non-SP cells and cardiomyocytes is concordant with their distinct phenotypes, and preferential clustering of Sca1^+^ cells with non-SP cells consistent with the predominance of non-SP cells in the Sca1^+^ population. Separation visualized by principal component (PC)2 and PC3 was attributable to four subsets of genes, which collectively define the main differences (*Myh6*, *Myl2*; *Gata4*, *Hand2*, *Tbx20*, *Tbx5*; *Cdh5*, *Kdr*; *Pdgfra*, *Tcf21*; Fig. 1d, below).

For most genes, expression or its absence was homogeneous within each respective population (Fig. 1b,c). However, cardiogenic genes were commonly bimodal in SP cells. Considering the functionally significant tetrad of *Gata4*, *Mef2c*, *Tbx5* and *Hand2* (ref. 30), only 8 of 43 cardiac SP cells expressed all four—a ‘mosaic' transcription factor phenotype in >80% of the cells. *Nkx2-5*, *Isl1* and *Hand1* were not detected. Of the cardiogenic genes identified, only *Mef2a* and *Mef2c* were expressed equivalently in SP and non-SP cells, each in a bimodal pattern (Fig. 1c). Thus, unlike cardiomyocytes, fresh single SP cells show highly mosaic expression of key cardiogenic genes, a potential block to their differentiation. Conversely, the existence of any cells with all four factors yet not target gene activation suggests that these factors do not suffice at their native levels to drive differentiation, or that other barriers exist.

Several stem cell-associated markers showed bimodal expression in SP and non-SP cells, equivalently, including ABC transporters (*Abcg2* and *Abcb1*) and two key genes for pluripotency (*Nanog* and *Pou5f1*); a third, *Klf4*, was present in nearly all Sca1^+^ cells regardless of SP status (Fig. 1c; Supplementary Fig. 1). Thus, differences in *Abcg2* and *Abcb1* mRNA do not establish the dye-effux phenotype—implicating potential differences in pump protein, activity or alternative transporters. Pluripotency genes showed no relation to the cardiogenic signature.

In summary, SP cells largely—but imprecisely—represent the adult cardiac Sca1^+^ cells having the molecular signature suggesting a cardiogenic phenotype. The dichotomous expression of *Pdgfra* and *Tcf21* versus *Cdh5* and *Kdr* defines more exactly the cells enriched for cardiogenic transcripts. Cardiac SP cells (more accurately, *Pdgfra*^+^/Sca1^+^ cells) express *Gata4/6*, *Mef2a/c*, *Tbx5/20* and *Hand2*, but rarely *Hand1*, *Isl1* and *Nkx2-5*, features of the first, second and both heart fields, respectively. Hence, these adult cells resemble a persistent but incomplete form of the developing cardiac mesoderm.

### Clonogenic self-renewing phenotype of cardiac Sca1^+^ SP cells

**Figure 2:**
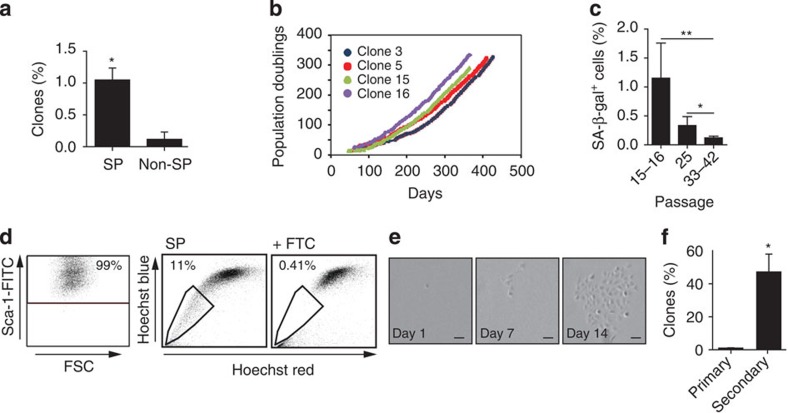
The Sca1^+^ SP phenotype identifies clonogenic, self-renewing cardiac cells. (**a**) Bar graph showing the % of adult cardiac clone-forming cells from Lin^−^ Sca1^+^ SP and non-SP fractions, after single-cell deposition. *n*=7; *P≤0.02. (**b**) Long-term growth of cloned cardiac SP cells. Clones shown are those used for grafting in Figs 3e and 4; Supplementary Fig. 4. (**c**) Lack of increased senescence-associated β-galatosidase (SA-β-gal) in four independent clones at increased passage number. *P≤0.02; **P≤0.0005. (**d**) Persistent Sca1 expression (left) and enrichment for the SP phenotype (right) in cardiac SP clones after long-term culture. Results shown here (clone 16 at passage 29) were confirmed in four additional clones. (**e**,**f**) Enrichment for 2° clone formation in cloned cardiac SP cells. (**e**) Serial bright-field images showing generation of 2° clone by a single cell from clone 3, passage 14. Scale bar, 100 μm. (**f**) 2° clone formation by six independent clones at passage 13–14. *P≤0.005. Data are shown as the mean±s.e.m. between independent experiments.

To pinpoint where the rare clonogenic capacity of Lin^−^/Sca1^+^ cells resides, and allow stringent production of single-cell derivatives, preparative flow sorting was performed. The direct cloning efficiency for single cardiac SP cells was ∼1% (21 clones/1,995 cells plated), in the absence of a feeder layer or prior adaptation to culture, ten-fold higher than for non-SP cells, analogous to the greater formation of colonies by SP cells in methylcellulose^12^ (Fig. 2a).

Cloned cardiac SP cells were propagated for >10 months and 300 doublings, without crisis or replicative senescence (Fig. 2b). At passage 15–16, <5% showed senescence-associated β-galactosidase activity; four clones were retested later, with 0.3% of cells positive at passage 25, indicating selection against this subpopulation (Fig. 2c). Clones even at 29 passages were highly enriched for Sca1 and the SP phenotype (Fig. 2d) and, at 14 passages, generated secondary clones with an efficiency >40% (6 primary clones tested, 1,425 cells plated; Fig. 2e,f). Thus, cloned cardiac SP cells are self-renewing and maintain their phenotype after long-term propagation.

### Cloned cardiac SP cells maintain the cardiogenic signature

**Figure 3:**
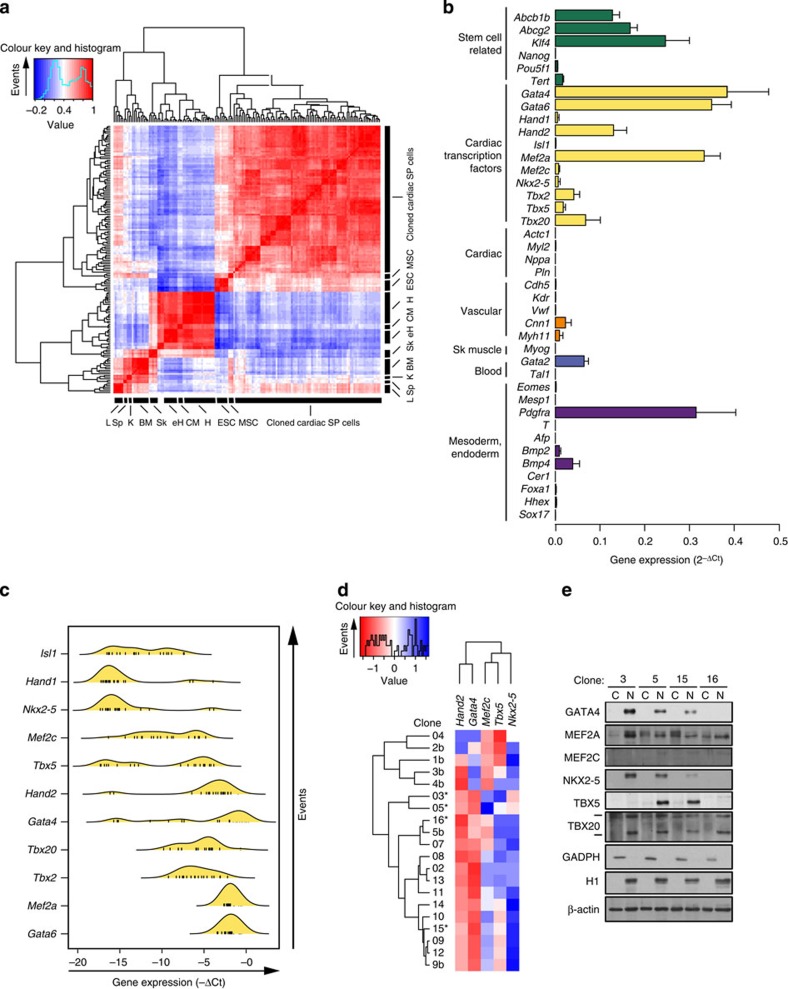
Fidelity of cloned cardiac SP cells to freshly isolated SP cells (**a**) Pearson correlation plot, each cell of the heatmap showing the sample pair's correlation coefficient (*r*). The clustering algorithm separates highly correlated samples (red: *r* close to 1) from weakly correlated ones (blue: *r* close to 0). The 20 independent lines of cloned cardiac SP cells show strong within-group correlation, moderate correlation to MSCs and ESCs, and weak correlation to other samples including heart. All clones were analysed at <30 passages. BM, bone marrow; CM, neonatal cardiomyocytes; eH, embryonic day 10 heart; ESC, undifferentiated AB2.2 ESCs; H, adult heart (encompassing whole heart, atria, and each ventricle); K, kidney; L, liver; MSC, PDGRα^+^ bone marrow MSCs; Sk, skeletal muscle; Sp, spleen. (**b**) Molecular signature of cloned cardiac SP cells. Bar graph, mean±s.e.m. for all 40 genes' expression in 20 independent clones. Genes are colour coded based on functional association or tissue specificity. For comparison with reference samples, see Supplementary Fig. 3. (**c**) Density plot showing the prevalence for expression of key cardiac transcription factors in the 20 clones. See Fig. 1d. The clustering algorithm separates genes with heterogeneous (multimodal) expression from those with homogeneous low or high expression. (**d**) Heatmap showing scaled expression of the four most heterogeneous transcription factors in the 20 clones (*Gata4*, *Mef2c*, *Tbx5*, *Hand2* and *Nkx2-5*). Expression within each sample is scaled to *Z*-score, 0 indicating mean expression of the four genes. Red indicates higher expression than the mean, while blue indicates lower expression. The two-dimensional hierarchical clustering algorithm orders the clones and genes based on co-expression profiles. Asterisks denote four clones taken forward to more detailed studies. (**e**) Western blots for cardiac transcription factors in the clonal lines. Cytoplasmic (C) and nuclear (N) fractions were analysed using glyceraldehyde 3-phosphate dehydrogenase and histone H1 to authenticate the fractions and β-actin as a loading control. The bands for TBX20 correspond to isoforms with or without the C-terminal 145 a.a. extension.

To learn whether cloned cardiac SP cells retain the cardiogenic transcription factors of the starting cells (Fig. 1b,c) or resemble the cCFU-F, lacking these^16^,^26^, we analysed >20 clonal cardiac SP lines for >40 relevant genes, using 384-well RT–PCR arrays (Fig. 3a–d). By hierarchical clustering of the Pearson correlation matrix, clonal cardiac SP cell lines were a contiguous entity distinct from PDGFRα^+^ bone marrow MSCs, undifferentiated ESCs, cardiomyocytes and reference tissues (Fig. 3a; Supplementary Fig. 3). Results are summarized as bar plots (Fig. 3b) and density plots depicting the prevalence of expression at each level (Fig. 3c). Cardiogenic transcription factors were enriched (Fig. 3b; Supplementary Fig. 3a). Expression was typically uniform across the clones, but variable or bimodal for several (*Gata4*, *Hand2*, *Mef2c* and *Tbx5/20*; Fig. 3c), as in fresh single SP cells (Fig. 1c). Most common were *Gata6*, *Mef2a* and *Tbx20*, with *Hand1*, *Isl1* and *Nkx2-5* least frequent (Fig. 3c). All but *Mef2c* were greater in cloned SP cells than in bone marrow MSCs (Supplementary Fig. 3a). Other features included: (i) enrichment for stem cell-associated markers (*Abcg2*, *Abcb1b* and *Klf4*), though not *Nanog* or *Pouf51*; (ii) no cardiomyocyte markers (*Actc1*, *Myl2*, *Nppa* and *Pln*); (iii) little or no expression of vascular (*Cdh5*, *Kdr*, *Vwf*, *Cnn1* and *Myh11*) and hematopoietic (*Tal1*) markers, excepting *Gata2*; and (iv) high expression of *Pdgfra*.

Given the presence of *Gata4*, *Mef2c*, *Tbx5*, *Hand2* and *Nkx2-5* in the ancestral network for cardiogenesis^31^, their importance to cardiogenic differentiation by ectopic transcription factors^30^,^32^, and their heterogeneous expression in fresh cardiac SP cells, these genes were plotted by two-dimensional clustering to visualize more clearly their state of co-expression in clonal progeny (Fig. 3d). Expression was highly mosaic, with permutations of 2–3 factors, but never all five simultaneously, and *Nkx2-5* being rarest. Hence, the clones recapitulate the single cells' phenotype of mosaic co-expression. Cardiac transcription factors were well maintained over time, apart from loss of *Tcf21* (Supplementary Fig. 3b,c). Given the existence of rare fresh cells co-expressing *Gata4*, *Mef2c*, *Tbx5* and *Hand2*, but never clones, such cells might be least clonable.

Thus, by single-cell qRT–PCR and clonal analysis, cardiac SP cells resemble an incomplete but stable form of heart-forming mesoderm, a transient phenotype in embryos and embryoid bodies. What maintains their arrested development is unknown. Possibilities include failure to express the transcription factors as nuclear proteins. Hence, western blotting was performed on nuclear and cytoplasmic lysates of four clones (indicated with * in Fig. 3d). TBX20 and MEF2A were detected in all, as expected from their mRNA expression (Fig. 3d), and were nuclear-localized (Fig. 3e; Supplementary Fig. 4). GATA4, TBX5 and NKX2-5 were nuclear in all clones expressing these and absent from the remainder (Fig. 3d,e). Weak or absent expression of *Mef2c* was paralleled by lack of MEF2C protein. For expressed genes, protein expression was highly uniform (>85% positive; Supplementary Fig. 5). Thus, cloned cardiac SP cells resemble the molecular signature of freshly isolated SP cells, and clonal variations recapitulate the fresh cells' microheterogeneity.

### Tri-lineage cardiovascular markers develop after grafting

Intact myocardium provides the best-agreed environment for differentiation of cardiac progenitors into cardiomyocytes and vascular cells^8^,^10^,^13^,^15^,^16^,^21^. To test whether cloned cardiac SP cells maintain their differentiation capacity and ascertain with single-cell progeny their multilineage potential, representative clones expressing mOrange were injected into the infarct border zone (clones 3, 5, 15 and 16). Cell retention 1 day later varied from 1 to 8%, declined to 0.1–0.5% at 2 weeks, and was no greater in control uninjured hearts. Retention ≤10% with later further loss is consistent with other studies^33^.

**Figure 4:**
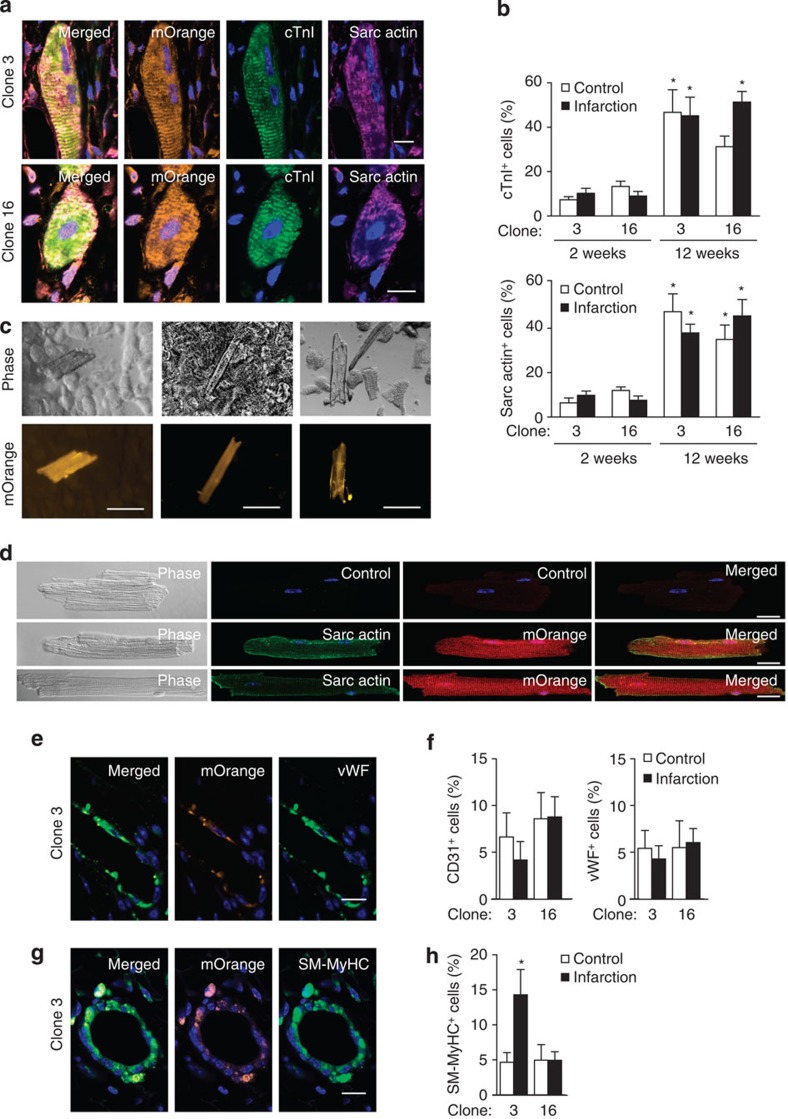
Cloned cardiac SP cells show tri-lineage potential after cardiac grafting. Clones were transduced with mOrange and delivered by intramural injection into mice subjected to sham operation (control) or coronary artery ligation (infarction). (**a**) Co-expression of cardiac troponin I (cTnI, green) and sarcomeric α-actin (sarc actin, violet) in striated grafted cells at 12 weeks. (**b**) Bar graph showing the proportion of mOrange^+^ cells expressing sarcomeric proteins at 2 or 12 weeks. (**c**,**d**) Cardiomyocyte isolation was performed on hearts injected with mOrange^+^ cloned cardiac SP cells at the time of infarction 12–14 weeks earlier. mOrange^+^ rod-shaped cardiomyocyte-like cells were detected in the preparations. (**c**) Representative cells from three independent hearts are shown. Scale bar, 100 μm. (**d**) Representative cells co-stained for mOrange^+^ and sarcomeric α-actin. Control, secondary antibody only. Scale bar, 20 μm. (**e**) Induction of von Willebrand factor (vWF, green) and localization of mOrange^+^ cells in vessels at 12 weeks. (**f**) Bar graph showing the proportion of mOrange^+^ cells expressing vWF at 2 weeks. (**g**) Induction of SM-myosin heavy chain (SM-MyHC, green) and localization of mOrange^+^ cells in vessels at 12 weeks. (**h**) Bar graph showing the proportion of mOrange^+^ cells expressing vWF at 2 weeks. At least 200 cells were scored for each clone for each condition, using at least three sections ≥80 μm apart containing mOrange^+^ cells. Data for each clone and time point are the mean±s.e.m. for 3–4 control hearts and 5–7 infarcted ones. Immunohistochemistry is shown here for two clones at 12 weeks and for all four clones at 2 weeks in Supplementary Fig. 4.

One day after grafting, no precocious expression was seen for seven markers of differentiation (Supplementary Fig. 6a). At 2 weeks, 10% of donor cells expressed cTnI and sarcomeric α-actin, of which half were double stained; similar results were obtained for myosin light chain 2v plus sarcomeric myosin heavy chains (Fig. 4b; Supplementary Fig. 6a, b). The differentiating mOrange^+^ cells were mononucleated and lacked organized sarcomeres, indicating immaturity.

By contrast, at 12 weeks, cTnI and sarcomeric α-actin were expressed in up to 50% of donor-derived cells, with organized sarcomeres and binucleation also detected (Fig. 4a,b). The mOrange^+^ cells detected histologically at this later time point were scarce, however, and only 5–10 mOrange^+^ rod-shaped cells were recovered from each heart following enzymatic dissociation at 12–14 weeks (Fig. 4c,d).

Endothelial markers CD31 and vWF were expressed in 4–8% of cells beginning within 2 weeks (Fig. 4e,f; Supplementary Fig. 6c,d), and SM-myosin heavy chains in 5–15% (Fig. 4g,h; Supplementary Fig. 6e, f). Although not easily identifiable within vascular structures after short-term grafting, donor-derived differentiating cells were found at 12 weeks in the endothelial and SM layers (Fig. 4e,g).

Overall, no difference was seen between injured and uninjured hearts, or between clones, except increased SM formation after infarction with one. Little or no staining was seen after injection of tail tip fibroblasts (Supplementary Fig. 6g).

**Figure 5:**
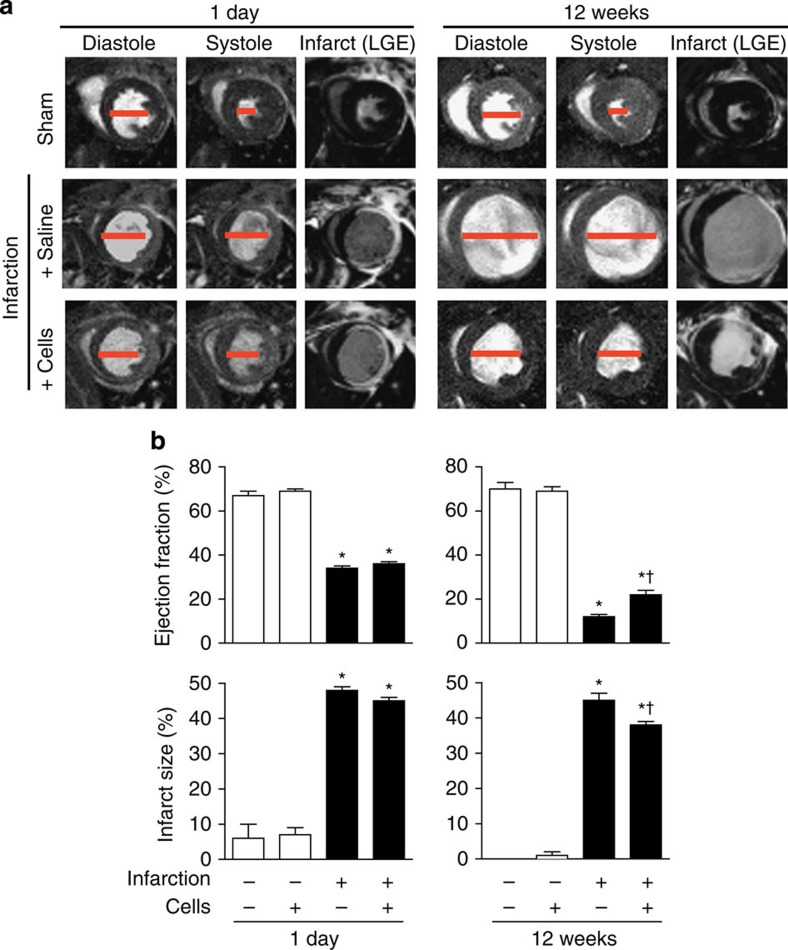
Intramyocardial delivery of cloned cardiac SP cells improves cardiac structure and function. Views by cine-MRI are shown at end-diastole and end-systole, along with late Gadolinium (Gd)-enhanced MRI (LGE) for infarct size. (**a**) Representative images of mouse hearts at 1 day and 12 weeks after infarction (MI) and grafting of clones. The red bar highlights end-systolic and end-diastolic diameters. (**b**) Serial measurements showing improved ejection fraction and diminished infarct size at 12 weeks in infarcted hearts that received cloned cardiac SP cells. Bar graphs present the mean±s.e.m. (sham+vehicle, *n*=4; sham+SP cells, *n*=10; MI+vehicle, *n*=8; MI+SP cells, *n*=20). *P<0.05 compared with the corresponding uninfarcted control hearts; ^†^*P*<0.05 compared with infarction plus the cell-free vehicle control injection.

To assess whether grafting-cloned SP cells had a functional impact following infarction, serial magnetic resonance imaging (MRI) measurements were performed (Fig. 5). At one day, no differences were seen between the infarcted groups with and without SP cells, both having similar infarct area and reduction of ejection fraction. Notably, at 12 weeks, cell grafting preserved ejection fraction (22±2%), compared with the vehicle-treated control (12±1%), reduced infarct size (38±1% versus 45±2%) and reduced the prevalence of severe left ventricle (LV) remodelling (LVEDV 3 × the control value) by 44% (*P*=0.02 by Pearson's *χ*^2^-test).

These data using single-cell clones support the tri-lineage potential of cardiac SP cells^24^, and suggest that their ‘developmental arrest' can be overridden despite incomplete expression of the cardiogenic programme, within the range of factors' represented here. Infarct size, LV function and the prevalence of severe remodelling all were improved by cell grafting. However, biomechanical benefits under these conditions of delivery are more plausibly due to paracrine effects (as in related publications^34^,^35^) than to direct actions of the infrequently persistent, newly formed mOrange-positive cardiomyocytes.

### Cardiac SP cells derive from Isl1- and Nkx2-5-expressing cells

**Figure 6:**
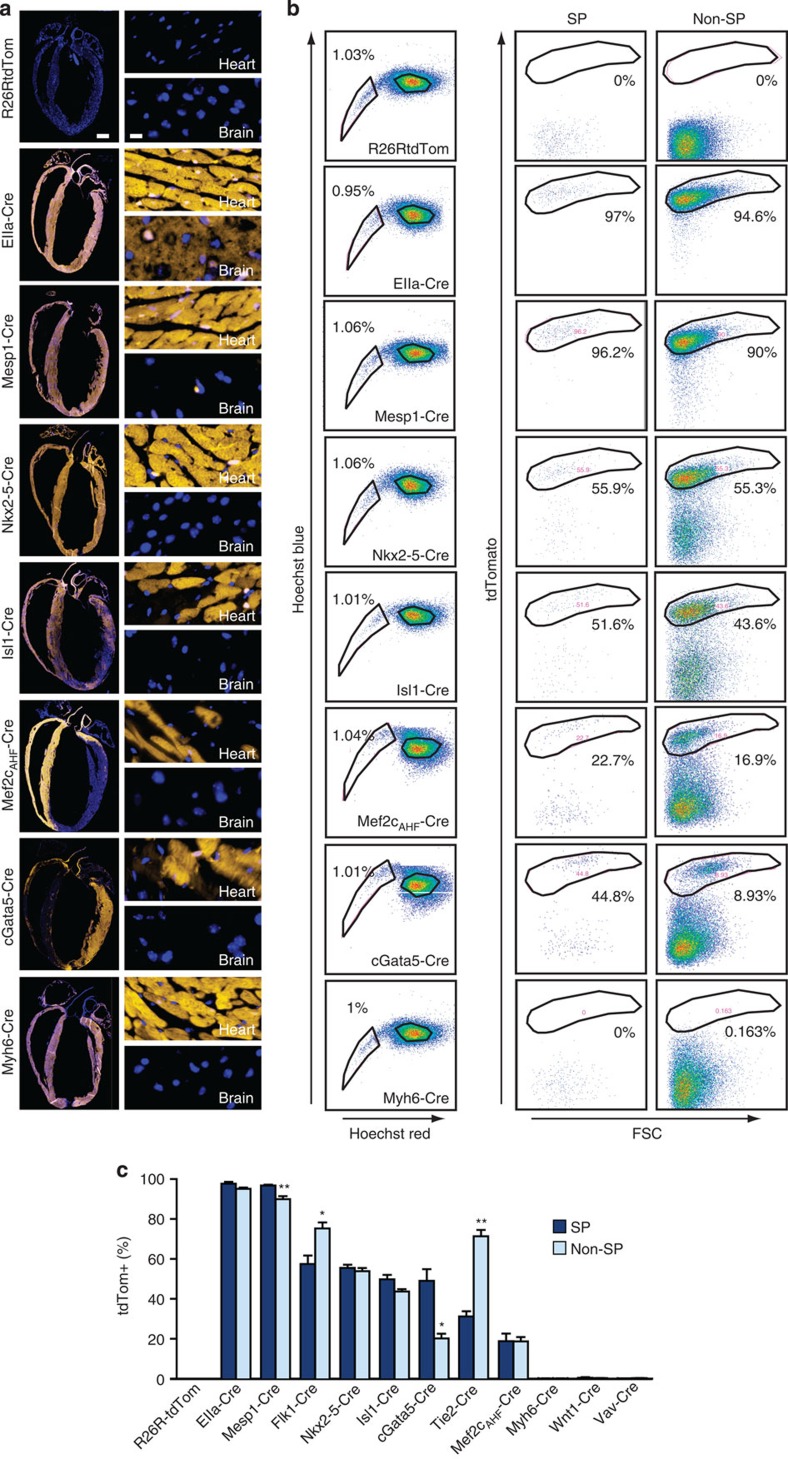
Sca1^+^ SP cells derive from *Mesp1-*, *Nkx2-5-*, *Isl1-*, *Gata5-* and *Wt1*-fated cells. (**a**) Cre drivers were crossed with the Ai14 R26R-tdTom line. Left, longitudinal sections of the heart at low magnification. Right, ventricular myocardium and brain at high magnification. Orange, Cre-dependent expression of tdTom; blue, 4′,6-diamidino-2-phenylindole. Scale bar, 1 mm (left), 25 μm (right). (**b**,**c**) Quantitation of cardiac SP and non-SP cells derived from the Cre^+^ ancestors shown. (**b**) Left, flow cytometry showing no interference of Cre with SP cell number. Right, tdTom in the SP and non-SP fractions of Lin^−^/Sca1^+^ cells. (**c**) Bar graph, % tdTom^+^ cells; *n*=3–8; *P≤0.05; **P≤0.001. Data are shown as the mean±s.e.m. for independent experiments.

Potential origins of cardiac SP cells include the specialization of primitive mesoderm denoted by *Mesp1*, a determinant of multipotent cardiovascular progenitor cells^36^. Like fresh adult cardiac Sca1^+^ cells^8^, fresh single SP cells and clones typically lack *Nkx2-5* and *Isl1* (Figs 1 and 3; Supplementary Figs. 1,3) yet could be the progeny of cells expressing these formerly, that is, after specification by *Mesp1*. To evaluate these and other possibilities, we performed Cre/lox fate mapping, converting antecedent expression of Cre recombinase into persistent activation of a sensitive reporter, the Ai14 form of *Rosa26-tdTomato* (tdTom)^37^. The fidelity of each bigenic line (Supplementary Table 3) was confirmed by induction of tdTom (Fig. 6a) and contributions to cardiac Lin^−^/Sca1^+^/SP cells were quantitated by flow cytometry (Fig. 6b,c). Cre driven by the viral EIIa promoter evoked ubiquitous tdTom. Without Cre, no recombination occurred. With *Mesp1-Cre*, most cardiac cells expressed tdTom, including myocytes, vessels and interstitial cells. Virtually all Lin^−^/Sca1^+^ cells derived from *Mesp1*^+^ precursors, whether SP or not, suggesting an origin from pre-cardiac mesoderm. Neither SP nor non-SP cells were derived from neural crest, hematopoietic cells or pre-existing cardiomyocytes, defined using *Wnt1-*, *Vav-* and *Myh6-Cre*.

*Nkx2-5*^+^ precursors generate most cardiomyocytes in the heart, plus endothelium and SM; *Isl1*^+^ progenitors give rise chiefly to the right ventricle, outflow tract and atria (from the second heart field), contributing less to the left ventricle (from the first heart field)^38^. In all chambers, as expected, most cells were *Nkx2-5*-fated (Fig. 6a). Left ventricular labelling was patchy with *Isl1*-*Cre*, whereas second heart field derivatives labelled uniformly. By flow cytometry, more than half the Lin^−^/Sca1^+^ cells were *Nkx2-5*-derived*—*fivefold more than for cCFU-Fs^16^—with an equal contribution from *Isl1*-fated cells (Fig. 6b,c). Less than 20% of SP and non-SP cells were fated by anterior heart field-restricted *Mef2c-AHF-Cre*. Whereas *Nkx2-5* and *Isl1* were expressed rarely in fresh single cells and cloned cardiac SP cells (Figs 1 and 3), their large contribution to the fate map suggests an origin from cardiac mesoderm, involving both heart fields or just the second. Alternatively, as *Nkx2-5* and *Isl1* contribute to the pro-epicardial organ^39^, a pro-epicardial origin is possible. Indeed, 50% of cardiac SP cells were labelled by proepicardium-expressed *cGATA5*-*Cre* (Fig. 6b,c), albeit less than in cCFU-Fs^16^. By contrast, non-SP cells showed greater contributions from *Flk1*- and *Tie2-Cre*, consistent with their endothelial lineage markers *Cdh5* and *Kdr*.

**Figure 7:**
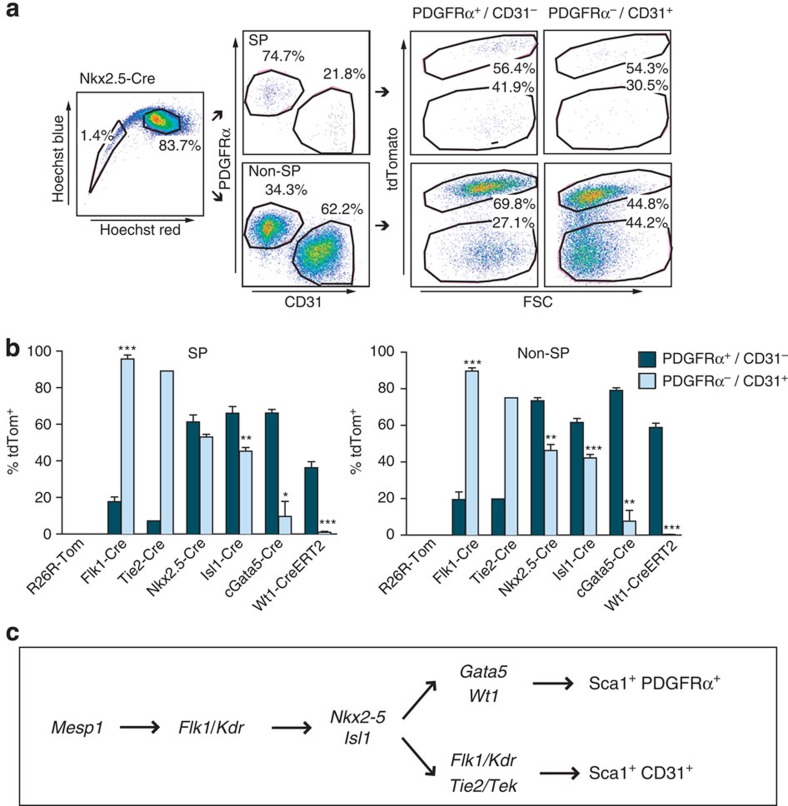
c*Gata5* and *Wt1* contribute specifically to PDGFRα^+^/CD31^−^ cells. (**a**) Flow cytometry illustrating the four-way strategy to dissect fate mapping in cardiac Lin^−^/Sca1^+^ cells by SP staining plus PDGFRα and CD31. Representative results are shown at the right for strong induction of tdTom in all four populations by antecedent expression of *Nkx2-5-Cre*. (**b**) Precursors expressing c*Gata5* and *Wt1* give rise to the PDGFRα^+^/CD31^−^ cells. Little or no difference was seen between SP and non-SP cells having the same PDGFRα/CD31 phenotype. Data are the mean±s.e.m. for tdTom induction by each Cre line in the four populations; *n*=2–7 excepting *n* =1 for *Tie2-Cre*; *P<0.05; **P<0.01; ***P<0.001. (**c**) Schematic representation of the origin of cardiac Sca1^+^ cells. The developmental stages and transitions shown are detailed in the text.

**Figure 8:**
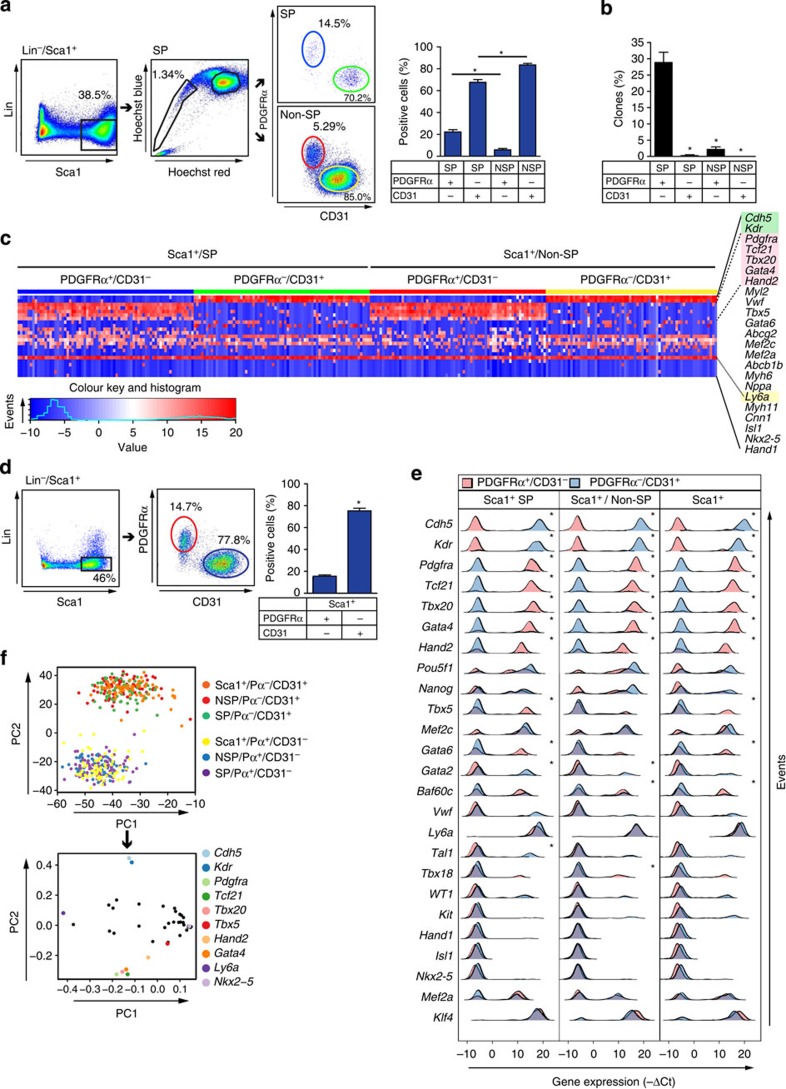
Precise co-segregation of the cardiogenic signature to PDGFRα^+^/CD31^−^ SP and non-SP cells. (**a**) Left, FACS density plots illustrating the four-way strategy to flow-sort Lin^−^/Sca1^+^ cells by SP staining plus PDGFRα and CD31. Right, bar graph of the subpopulations' prevalence. Mean±s.e.m.; *n*=5; *P<0.0001. (**b**) Bar graph indicating the % of single cells generating clones. Mean±s.e.m.; *n*=4–5; *P<0.0001. (**c**) Single-cell qRT–PCR profiles, showing co-expression of selected genes in Pdgfrα^+^/CD31^−^ versus Pdgfrα^−^/CD31^+^ cells from the SP and non-SP fractions. The heatmap shows expression as −ΔCt values (blue, low; red, high). Genes are ordered based on the variance of mean expression levels of the four groups. Highlighted genes: green, *Cdh5* and *Kdr*, enriched in PDGFRα^−^/CD31^+^ cells; light red, *Pdgfra*, *Tcf21*, *Tbx20*, *Gata4* and *Hand2*, in PDGFRα^+^/CD31^−^ cells; yellow: Ly6a, in all four subpopulations. *n*=60–70 for each. (**d**) Left, gating strategy to identify mutually exclusive PDGFRα^+^ and CD31^+^ cells within the Sca1^+^ population. Right, bar graph showing the % of cells from the indicated populations. Mean±s.e.m.; *n*=5; *P<0.0001. (**e**) Density plots show −ΔCt values of the indicated genes in single PDGFRα^+^/CD31^−^ (light red) and PDGFRα^−^/CD31^+^ (light blue) cells from the three sample groups (SP, non-SP and Sca1^+^). Genes are ranked based on the loadings extracted from PC2, which reflect the across-sample variability. (**f**) PCA of the single-cell expression profiles from the six populations in panel **e**. (Top) PC1 (43% of variability) captures within-group variance. PC2 (20% of variability) corresponds to between-group variance, mainly separating cells according to PDGFRα/CD31 status, irrespective of their isolation as SP, non-SP or total Sca1^+^ cells. (Bottom) Gene loadings associated with PC1 and PC2. Genes associated with PC1 are unrelated to cell class: consistently absent/low expression across all samples at the left (*Nkx2-5*) and high expression at the right (*Ly6a*). PC2 resolves the genes expressed in different cell populations: *Cdh5* and *Kdr*, prevalent in PDGFRα^−^/CD31^+^ cells, at the top, with *Pdgfra*, *Tcf21* and cardiac transcription factors, prevalent in PDGFRα^+^/CD31^−^ cells, at the bottom.

Given that differences between cardiogenic and endothelial lineage genes in SP and non-SP cells mapped to the *Pdgfra*^+^ and *Cdh5*^+^/*Kdr*^+^ phenotypes, respectively, we refined the Cre/lox analysis, adding antibodies against PDGFRα and the endothelial lineage marker CD31. SP and non-SP cells were each divided into two subpopulations, PDGFRα^+^/CD31^−^ and PDGFRα^−^/CD31^+^ (Fig. 7). These two features can be mutually exclusive^16^,^40^ or partially overlapping (Fig. 7a; cf. Fig. 8a), hence their combined use for prospective sorting is more stringent than PDGFRα alone. Regardless of the SP phenotype, ∼90% of PDGFRα^−^/CD31^+^ cells were labelled by *Flk1-Cre*, unsurprising given this fraction's expression of *Flk1/Kdr.* By comparison, *Flk1-Cre* and *Tie2-Cr*e contributed little to the PDGFRα^+^/CD31^−^ SP and non-SP cells (10–20%).

Conversely, PDGFRα^+^/CD31^−^ cells were highly labelled by *cGata5-Cre* and by activating *Wt1-CreERT2* with tamoxifen at E10.5 (70–80% and 40–60%, respectively). Tamoxifen at 8 weeks labelled none of the four Sca1^+^ populations. PDGFRα^−^/CD31^+^ cells were infrequently labelled by *cGata5-Cre* (10%), with no contribution from embryonic activation of *Wt1-CreERT2*; this may reflect lesser sensitivity of CreERT2, especially at permissible doses of tamoxifen. Small differences were seen using *Nkx2.5-* and *Isl1-Cre*: 60–70% of PDGFRα^+^/CD31^−^ cells versus 40–50% of PDGFRα^−^/CD31^+^ ones.

These results are summarized in Fig. 7c, integrated with relevant aspects of cardiac origins during embryogenesis and ESC differentiation^41^,^42^,^43^,^44^,^45^,^46^. Virtually all cardiac Sca1^+^ cells, whether SP or non-SP, PDGFRα^+^ or CD31^+^, derive from *Mesp1*^+^ mesoderm. *Flk1*^+^ multipotent progenitors (likely co-expressing PDGFRα^46^,^47^) have potential to differentiate into cardiovascular lineages including cardiomyocytes, endothelial and SM cells. Adult PDGFRα^+^/CD31^−^ and PDGFRα^−^/CD31^+^ cells derive largely from *Nkx2-5*^+^ and *Isl1*^+^ precursors—with known cardiovascular tri-lineage potential^14^—genes whose contribution here concurs with their role in proepicardium development^38^, upstream of *Gata5* and *Wt1* (ref. 39). Incomplete labelling of PDGFRα^+^ cells by *Flk1-Cre* is likely due to lower or transient expression of *Flk1* relative to CD31^+^ cells^48^. That cardiac CD31^+^ cells were only rarely labelled by *cGATA5-Cre* and *Wt1-CreERT2* agrees with evidence that coronary endothelium derives from a *Wt1*^−^ precursor^49^. These divergent, antithetical subpopulations expressing PDGFRα (and cardiogenic genes) versus CD31 (and endothelial genes) associate imprecisely with SP status.

### PDGFRα tracks specifically with the cardiogenic signature

Given that fresh Lin^−^/Sca1^+^/SP cells and their clonal derivatives are enriched for *Pdgfra*, and that Sca1 and PDGFRα are attributes of MSC-like cardiac progenitors identified by the CFU-F assay^16^, PDGFRα and MSC markers were analysed by fluorescence-activated cell sorting (FACS; Supplementary Fig. 7). CD105 and CD90 were expressed in ≥70% of fresh cardiac SP cells and equally in non-SP cells. CD73, CD44 and PDGFRα were detected in just 20–40% of fresh cardiac SP cells, but two- to three-fold more often than in non-SP cells. Greater labelling for PDGFRα concurs with SP cells' greater expression of *Pdgfra*.

Consequently, we performed four-way preparative sorting much as done for Fig. 7: cardiac Lin^−^/Sca1^+^ cells were partitioned on the basis of SP versus non-SP phenotype, plus PDGFRα^+^/CD31^−^ versus PDGFRα^−^/CD31^+^ (Fig. 8a). The latter two dichotomous groups constituted >85–90% of the cells, with few double-positive or -negative cells. The yield by this approach was ∼2,400,000 Sca1^+^ cells/heart, with 10,000 PDGFRα^+^/CD31^−^ cells from the SP fraction and 90,000 from the non-SP fraction. (Although comprising a small minority of the non-SP cells, PDGFRα^+^/CD31^−^ non-SP cells outnumber the PDGFRα^+^/CD31^−^ SP cells, given the much rarer SP phenotype.)

### PDGFRα and the SP phenotype synergize for clonal growth

Next, the relative importance of PDGFRα, CD31 and the SP phenotype was compared in clonogenicity assays. Using ‘physiological hypoxia' to lessen hyperoxic stress and enhance clone formation^50^, the SP phenotype was dispensable for direct cell cloning (2.2±0.7% in PDGFRα^+^/CD31^−^/non-SP cells; 0±0% in PDGFRα^−^/CD31^+^/non-SP cells) but highly synergistic with the PDGFRα/CD31 phenotype (28.5±3.2% in PDGFRα^+^/CD31^−^/SP cells; 0.3±0.3% in PDGFRα^∼^/CD31^+^/SP cells; Fig. 8b). Proportionally similar differences were seen in normoxia, at lower rates of clone formation (12.6±1.3% in PDGFRα^+^/CD31^−^/SP cells; 0.5± 0% in PDGFRα^∼^/CD31^+^/SP cells; 1.6±0.96% in PDGFRα^+^/CD31^−^/non-SP cells; 0% in PDGFRα^−^/CD31^+^/non-SP cells).

Single-cell qRT–PCR as in Fig. 1 was performed on 60–70 cells from each of the four freshly sorted populations (Fig. 8c). *Gata4*, *Mef2c*, *Hand2* and *Tbx5/20* were enriched in PDGFRα^+^/CD31^−^ cells regardless of their SP status (all cells *Ly6a*^*+*^/*Pdgfra*^*+*^/*Tcf21*^*+*^). Conversely, *Cdh5* and *Kdr* were expressed in both PDGFRα^−^/CD31^+^ fractions (all cells *Ly6a*^*+*^/*Pdgfra*^−^/*Tcf21*^−^). Thus, single-cell profiles define two mutually exclusive subpopulations: one expressing *Pdgfra*, *Tcf21* and cardiac transcription factors (the phenotype of cloned cardiac SP cells), *Pdgfra*^−^ *cells* lacking these and having vascular markers instead.

### Prospective sorting of cardiogenic cells using PDGFRα

Because the SP phenotype was dispensable for clonogenicity and requires a mutagenic dye that is unsuited to translation, we tested the alternative that separating Lin^−^/Sca1^+^ cells based on PDGFRα^+^/CD31^−^ (omitting Hoechst 33342) would prospectively purify cells with a consistent cardiogenic signature (Fig. 8d–f). The single-cell profiles of all six populations—the PDGFRα^+^/CD31^−^ versus PDGFRα^−^/CD31^+^ subsets of Sca1^+^, SP and non-SP cells respectively—were compared with PCA (Fig. 8e). PDGFRα^+^ and PDGFRα^−^ populations were separated clearly, attributable to antithetical expression of *Pdgfra/Tcf21* versus *Cdh5/Kdr*, the former genes co-segregating with *Gata4/6*, *Hand2*, and *Tbx5/20*. Density histograms showed equivalent impact of PDGFRα on gene expression regardless of SP status (Fig. 8f). The ‘hinge' population between the rigorous SP and non-SP gates also was enriched for PDGFRα^+^/CD31^−^ cells, and cardiogenic gene expression here likewise tracked with PDGFRα (Supplementary Fig. 8). Conversely, gene enrichment in PDGFRα^−^/CD31^+^ cells included *Tal1 and Gata2*, hemangioblast transcription factors that, respectively, repress the cardiac fate and assist the binding of Tal1^51^.

Given the cardiogenic gene profile of PDGFRα^+^/CD31^−^ cells across all the various Sca1^+^ populations, we sought to test whether PDGFRα and CD31 might suffice for prospective sorting of cells capable of cardiac differentiation *in vivo*, as reported for CD31^−^ SP cells if co-cultured with cardiomyocytes^12^. PDGFRα^+^/CD31^−^ Sca1^+^ cells were purified as in Fig. 7d, expanded for ≤10 passages as was essential to produce a sufficient number for injection, and were then delivered to recipient hearts, with and without infarction. In both settings, engraftment and co-expression of α-sarcomeric actin were confirmed at 2 weeks (Supplementary Fig. 9). The reciprocal PDGFRα^−^/CD31^+^ population could not be tested under comparable conditions, however, as these do not undergo equivalent expansion.

## Discussion

For a decade, investigations of adult cardiac progenitor/stem cells have proposed diverse criteria to define and purify these cells and, recently, progress them into human trials of cardiac repair^19^. The relationships among reported cardiac progenitor cells remain frustratingly elusive, even among various Sca1^+^ cells. By microarray profiling of cardiac Sca1^+^, SP and c-kit^+^ cells, Sca1^+^ cells were closest to cardiomyocytes in their transcriptome-wide molecular phenotype and c-kit cells most remote^52^. Gaps in the present understanding of cardiac stem/progenitor cells may largely arise from molecular heterogeneities that can be unmasked using single-cell expression profiles as a guide. Such evidence would be essential to develop a dendrogram relating the potential cardiogenic reservoirs in adult myocardium.

Here we combined single-cell qRT–PCR profiling with preparative flow sorting, single-cell deposition, clonogenic assays and systematic investigation of the resultant clones to pinpoint Sca1^+^ cells that were enriched for the attributes of a cardiac stem cell. We specifically link *Pdgfra* with the cardiogenic signature of *Gata*, *Hand* and *Tbx* genes, features mutually exclusive with the endothelial markers *Cdh5* and *Kdr* in *Pdgfra*^−^ cells. This relation in turn led us to the PDGFRα^+^/SP cell as remarkably enriched for the cardiogenic gene signature and clonogenicity, the latter being not only just an attribute of ‘stemness' but also instrumental to prove multilineage potential after grafting.

Our results provide clarity into the intricate relationships among at least those cardiac progenitor cells with Sca1 in common, unmasking subpopulations and microheterogeneities previously unknown: (i) fresh single SP cells express the pluripotency markers *Nanog*, *Oct4* and *Klf4*, in concert with cardiac transcription factors; (ii) their cardiogenic signature is incomplete, resembling a *forme fruste* of cardiogenic mesoderm during embryogenesis; (iii) cardiac SP cells are highly enriched for *Pdgfra* mRNA and protein; (iv) *Pdgfra* and PDGFRα demarcate the cells expressing cardiac transcription factors with greater sensitivity and precision than does the SP phenotype; (v) the inverse correlation between cardiac and endothelial lineage genes suggests a mechanistic basis for differences in SP cells expressing or lacking CD31^12^; (vi) clonal progeny faithfully recapitulate the cardiogenic signature of the starting SP cells, including mosaic expression of *Gata*, *Hand* and *Tbx* factors; and (vii) PDGFRα is sufficient to define clonogenic cells prospectively, with ten-fold synergy for clonal growth in cells also possessing the SP phenotype. In physiological O_2_, the direct cloning efficiency of fresh single PDGFRα^+^/SP cells was>25%.

The consensus signature of cardiac PDGFRα^+^/Sca1^+^ cells is the presence of *Gata4/6*, *Mef2a/c*, *Tbx5/20* and *Hand2*, genes essential for normal cardiogenesis^31^,^53^ and encompassing four (*Gata4*, *Mef2c*, *Tbx5* and *Hand2*) whose supraphysiological expression drives fibroblasts to a cardiomyocyte-like phenotype^30^. Fresh single SP cells, like single clones, chiefly express just 2–3 of these, unsuspected heterogeneity we unmasked by single-cell profiling. Conceivably, such mosaicism might function to restrain precocious differentiation and exhaustion of the stem cell pool. While the existence of even a few cells co-expressing all four factors without their targets might seem paradoxical, it would be misleading to assume that the four suffice for cardiogenesis at native levels. Our results do not exclude heterogeneity in genes not measured, or epigenetic and post-translational impediments.

The cardiac SP cell produced by preparative cell sorting partially resembles the cCFU-F produced by colony formation (sharing clonogenicity, multilineage potential, co-expression of PDGFRα with Sca1), but also differs significantly, as the cCFU-F lacks enrichment for the cardiogenic genes emphasized here^16^,^26^. This may reflect differences in cell selection, if the colony-forming assay discerns or elicits a more primitive cell. Most aspects of the fate map were similar, both derived from mesodermal cells expressing *Mesp1*, and at least in part from the pro-epicardial organ based on *cGata5* and *Wt1*. Innately, Cre-lox fate-mapping can define only the minimal derivation from precursors expressing a given gene, contingent on the duration and level of Cre expression and on reporter gene functionality in a given context^39^. The few fate-mapping differences between freshly isolated cardiac PDGFRα^+^/Sca1^+^ cells and the cultured cCFU-F—chiefly, greater derivation from *Nkx2-5*^+^ cells—are quantitative, not qualitative. Biological distinctions are possible, consistent with the gene profiles, but technical differences in recombination are an alternative and perhaps more parsimonious interpretation. As fate mapping does not exclude dual origins from the cardiac crescent and pro-epicardial organ, it would be intriguing to compare further the *Nkx2-5*- and *Wt1*-fated cells.

Notably, the SP phenotype was largely but incompletely specific as a predictor of cardiogenic gene expression in single cells, whereas *Pdgfra* co-segregated with cardiac transcription factors with absolute precision. *Tcf21*, a feature of cardiac SP cells^11^, was strictly co-expressed in the *Pdgfra*^+^/PDGFRα^+^ cells enriched for heart-forming factors. *Tcf21*-fated cells contribute to the epicardium, coronary vessels and interstitium of the adult heart^54^. It is unknown whether *Pdgfra* and *Tcf21* function in adult cardiac Sca1^+^ cells' clonal growth and cardiovascular differentiation. Assuming a human counterpart exists for Sca1^+^/PDGFRα^+^ cells, we speculate that PDGFR may be useful towards obtaining cardiogenic cells from adult human hearts without need for the mutagen Hoecsht 33342 or selection in culture. PDGFRα^+^ progenitor cells exist in human hearts, whose capacity for cardiac differentiation is not settled^17^,^40^, and supplemental markers may be required. At the least, PDGFRα provides a rational means, in mice, to purify fresh adult cardiac cells that are uniformly enriched for cardiogenic genes, in greater yield and homogeneity than achievable with SP cells, as an enhanced platform for ‘omic' studies and the search for developmental catalysts.

## Methods

All animal procedures were performed under UK Home Office approval (PL 70/6806, 70/7880).

### Cell isolation and flow sorting

Adult male 8- to 13-week-old C57BL/6 mice (Charles River) were used for cell purification, analysis and cloning. Lines used for flow cytometry in conjunction with fate mapping are detailed in Supplementary Table 3. For activation of *Wt1-CreERT2 in utero*, tamoxifen was given to gestating females at E10.5 as a single dose of 0.04 mg g^−1^ (1 mg per mouse), reduced from 0.12 mg g^−1^ (ref. 55) and with the addition of progesterone (1 mg per mouse)^16^ to improve embryo survival. For recombination in adult mice, tamoxifen was given at 8 weeks for 5 days consecutively (2 mg per day per mouse). Tamoxifen and progesterone were diluted in corn oil and administered by gavage.

Hearts were harvested, minced and enzymatically dissociated using 100 μg.ml^−1^ Liberase TH Research grade and 50 μg ml^−1^ DNAse I (Roche Applied Science), with four to five cycles of digestion for a total maximum of 45 min at 37 °C. The resulting cardiomyocyte-depleted cell preparation was filtered through 70 μm nylon mesh (BD Falcon). Hematopoietic lineage (Lin) depletion and Sca1 enrichment were performed by immunomagnetic separation (AutoMACS Pro Separator, Miltenyi Biotec). For Lin depletion, a cocktail of biotinylated antibodies was used with super-paramagnetic microbeads (Miltenyi Biotec). For Sca1 enrichment, cells were subsequently labelled with anti-Sca1-FITC antibody and anti-FITC microbeads (Miltenyi Biotec), then purified by four cycles of positive selection. In fate-mapping analyses, to minimize cell loss before flow sorting, no Sca1 enrichment was performed. To resolve SP and non-SP cells, cells were stained with 5 μg^ ^ml^−1^ Hoechst 33342 (Sigma-Aldrich) and filtered as above. Control samples for the dye-efflux assay included Hoechst 33342 with one or more of the following inhibitors: 10 μM fumitremorgin C (Merck), 50 μm verapamil (Sigma-Aldrich) or 5 μm reserpine (Sigma-Aldrich). The final step comprised staining with antigen-presenting cell (APC)-conjugated streptavidin (eBioscience), to resolve any residual contaminating Lin^+^ cells. Propidium iodide was added as the routine dead cell marker. For fate-mapping analyses, TO-PRO3 was used instead, for compatibility with the tdTom reporter; for *cGATA5-Cre* and *Mef2c-AHF-Cre*, TO-PRO5 was used for additional compatibility with APC. Where indicated, other minor modifications were made for specific experiments.

For isolation of neonatal mouse cardiomyocytes (1–3 days old), the hearts were placed in Ca^2+^- and Mg^2+^-free phosphate-buffered saline containing 10 g l^−1^ D-glucose (PBS–Dg), washed 3 × in PBS–Dg and minced to obtain fragments of <1 mm. This was followed by enzymatic dissociation in PBS–Dg containing 0.2% collagenase type 2 (∼50 U mg^−1^), 0.05% DNAse (∼3,200 U mg^−1^) and 0.05% Trypsin (∼250 U mg^−1^; #4179, #2009 and #3707, respectively; Worthington). Digestion was performed for five rounds × 10 min each with agitation at 37 °C. At the end of each round, dissociation was facilitated by titruation and the dissociated cell supernatant was transferred to D-MEM/F-12 media containing GlutaMAX, 50 mg ml^−1^ gentamicin (both, Invitrogen) and 10% fetal bovine serum (Hyclone). The enzymatic solution was replaced with fresh solution for each round^56^. At the end of the mechanical and enzymatic dissociation, myocytes were collected by centrifugation, followed by Percoll purification using 1.05, 1.06 and 1.82 g ml^−1^ densities at 2,000*g* for 30 min. The myocyte-enriched fraction (interface between the 1.06 and 1.082 g ml^−1^ layers) was harvested, washed and further enriched by differential plating on standard tissue culture plates, upon which the cardiac fibroblasts adhere rapidly. After 45 min, the myocyte-enriched suspension was collected and redeposited onto Primaria tissue culture plates in D-MEM/F-12 containing 5% horse serum (Hyclone). Tail tip fibroblasts were obtained by mechanical and enzymatic dissociation, using 0.25% Trypsin–EDTA at 37 °C for 30 min with continuous rocking. PDGFRα^+^ Sca1^+^ CD45^−^ TER119^−^ bone marrow MSCs were kindly provided by S. Rankin^57^.

The FACSAriaII flow sorter and LSRII flow cytometer (Becton Dickinson), optimized for use in the Hoechst 33342 assay, were equipped identically with 355 nm ultraviolet, 405 nm violet, 488 nm blue, 561 nm yellow–green and 638 nm red lasers. FlowJo vX was used for data analysis (versions 9.3.1 and 10.0.06; Tree Star).

### Cell cloning and robotic cell culture

Clones were generated by single-cell deposition (FACSAriaII) into standard 96-well plates coated with 50 μg ml^−1^ collagen type I (BD Bioscience). Where indicated, pools of 500–2,000 cells were each seeded into a well of 96-well plates. Cells were cloned and propagated in medium containing 35% Iscove's Modified Dulbecco's Medium, 32.5% Dulbecco's Modified Eagle's Medium, 32.5% Ham's F12 (all Invitrogen), 3.5% bovine growth serum (Hyclone), 1.3% B27 supplement (Invitrogen), 6.5 ng ml^−1^ recombinant human epidermal growth factor, 13 ng ml^−1^ recombinant human fibroblast growth factor-basic (154 a.a., Peprotech), 0.0005 U ml^−1^ thrombin (Roche), 0.65 ng ml^−1^ CT-1 (Cell Sciences), 2 mM L-glutamine (Invitrogen), 1 × Antibiotic-Antimycotic (Invitrogen) and 0.14 mM 2-mercaptoethanol (Sigma-Aldrich)^15^. Clone formation was defined as a colony ≥1,500 μm in diameter (usually >500 cells). Cells were trypsinized and expanded upon reaching 70–80% confluency. Except where indicated, cells were maintained at 37 °C in ambient O_2_ (21%) and 5% CO_2_.

Following initial pilot studies done manually, clone generation and propagation were performed using a customized Industrial Robot Integrated System (Beckman Coulter), comprising two Cytomat24C cell culture incubators with capability for hypoxic conditions (Thermo Fisher), compact HP 3JC Motoman robotic arm (Yaskawa), Cytomat Microplate Hotel (Thermo Fisher), Biomek FXP liquid handling system (Beckman Coulter), CloneSelect Imager (Molecular Devices) to evaluate confluency, Vi-CELL XR cell viability analyser (Beckman Coulter) for trypan blue dye exclusion assays, and Bigneat enclosure. Secondary clones were plated as above. Senescence-associated beta-galactosidase activity was determined, using the Abcam Senescence Detection Kit.

### Quantitative RT–PCR

mRNA was extracted manually using TRI reagent or robotically using the Agencourt RNAdvance Tissue system and a Biomek FXP liquid handler (Beckman). Samples were treated with TURBODNase (Applied Biosystems) and RNA quantitated using a Nanodrop 8000 Spectrophotometer (Thermo Fisher Scientific). Reverse transcription was performed with 0.25–1 μg of RNA using the High Capacity cDNA kit (Applied Biosystems). qRT–PCR was performed using TaqMan primer/probe sets (Supplementary Table 2), customized TaqMan low-density array cards and an ABI PRISM 7900HT Sequence Detection System (Applied Biosystems). Cycle number at threshold was calculated using RQ Manager 1.2.1 (Applied Biosystems).

Data for TaqMan low-density array cards and single-cell qRT–PCR were processed by the ΔCt method^58^, in the R/Bioconductor platform (version 2.15.2) using custom R code. The heatmaps and density plots were visualized using the *gplots* (CRAN repository) and *beanplot* packages, respectively.

For the analyses of cardiac SP clones in comparison to a selection of reference tissues (Fig. 3, Supplementary Fig. 2), the expression of 40 test genes was measured together with 3 ubiquitous normalization controls (*18 S*, *Hmbs* and *Ubc*) for each sample. A total of 20 independent clonal cardiac SP lines was investigated, at varying passage number <30 (40 groups) and including replicated samples (for a total number of 87). The expression of each sample was normalized to the combined expression (geometric mean) of the two most stable reference genes (*Hmbs* and *Ubc*) to obtain the normalized value of ΔCt^59^.

The correlation between the cardiac SP clones and reference tissues was evaluated by calculating the Pearson correlation (*r*) for all sample pairs from the ΔCt values for all 40 test genes for each sample. Results were then arranged by hierarchical clustering and visualized as a heatmap (Fig. 3a). The Pearson correlation (*r*) can extend from −1 to 1. A value close to 1 indicates a high positive correlation (all genes expressed similarly between the two samples), close to 0 (blue) a weak correlation and close to −1 an anti-correlated gene expression profile.

The molecular signature of the cardiac SP clones is shown as bar plots on a linear scale (2^−ΔCt^) in Fig. 3b, which shows the mean expression of all 40 measured genes in the 20 clones. The genes were grouped and colour coded on the basis of functional association and tissue specificity. The *nparcomp* R package was used to perform nonparametric multiple comparisons based on relative contrasts effects. Dunnett's contrasts were specified, where each reference tissue is compared with the cloned SP samples (Supplementary Fig. 2). The statistic was then computed using multivariate transformation (Probit transformation function).

To elucidate the expression profile of key cardiac transcription factors across the single cardiac SP clones, inverted ΔCt values for each gene were visualized in a density plot (Fig. 3c). To estimate the population density of expression for each gene, we used a Gaussian kernel density estimation procedure applied to each of the 20 SP clones (short vertical lines). Again, the genes were ordered using hierarchical clustering. During this process, the expression of cardiogenic transcription factors within each sample was first scaled by their mean and s.d. This results in a *Z*-score, with zero mean and unit variance, which normalizes the sample-specific variability. Subsequently, the clustering algorithm was applied to separate genes with heterogeneous (multimodal) expression (*Gata4*, *Mef2c*, *Tbx5* and *Hand2*) from genes with homogeneous low/negative expression (*Isl1*, *Hand1*and *Nkx2-5*) and homogeneous high expression (*Tbx20*, *Tbx2*, *Mef2a* and *Gata6*).

### Single-cell qRT–PCR

Single cells were sorted directly (FACSAria II) into 96-well plates containing 10 μl of the reaction mixture for pre-amplification, using CellDirect One-Step qRT–PCR Kits (Invitrogen). Pre-amplification was performed in a Veriti Thermal Cycler (Applied Biosystems) for 22 cycles. As negative controls, at least 3–5 non-template samples were included in each run at the pre-amplification stage. Quantitative amplification was performed using Dynamic Array chips for 48 assays × 48 samples, the BioMark HD system (Fluidigm) and TaqMan primer/probe sets as above. The stability of endogenous controls was estimated^59^ and each sample was consequently normalized to ΔCt using the expression of *Ubc*. Samples were centred around their individual means by subtracting the mean expression of all the genes within the sample. This results in a standardized gene expression with zero mean in each sample, thus correcting the technical error associated with batch acquisition.

To visualize the expression of key genes in the populations tested, data were plotted in colour-coded heatmaps of inverted ΔCt values (Figs 1 and 8): blue (gene expression low/none) to red (high). Samples were ordered on the basis of cell type, and the genes grouped by a hierarchical clustering algorithm according to the underlying co-expression pattern (Fig. 1) or variance between the sample classes (Fig. 8). Individual gene expression levels between selected samples were compared using density plots. To identify differential expression, the genes were classified as present or absent using a Gaussian mixture model classification procedure. The frequency distribution of each gene was then calculated from the combination of two sample classes: SP versus non-SP (Fig. 1) or PDGFRα^+^/CD31^−^ versus PDGFRα^−^/CD31^+^ (within SP, non-SP and Sca1+ groups; Fig. 8). The level of significance was obtained with Fisher's Exact test and the resulting *P*-values adjusted using the Bonferroni correction.

Differences between samples were investigated using PCA^60^. PCA applies multiple linear transformations (singular value decomposition) to the expression profiles (standardized ΔCt values) of individual samples and identifies a series of PCs that elucidate the most distinguishing features between the samples. The linear projections (PC scores) attempt to maximize the variation between the samples, whereas the coefficients of those projections (PC loadings) measure the importance of genes in defining the underlying variability associated with each component.

### Western blotting

For western blotting, cells and tissues were lysed for 45 min at 4 °C in radioimmunoprecipitation assay (RIPA) buffer (Sigma-Aldrich), with the addition of cOmplete, EDTA-free, Protease Inhibitor Cocktail and PhosSTOP Phosphatase Inhibitor Cocktail tablets (Roche). Nuclear and cytoplasmic lysates was isolated using Nuclear/Cytosol Fractionation kits (Biovision). Proteins were quantitated by the Pierce BCA assay kit (Thermo Fisher). Lysates were size-fractionated by SDS–polyacrylamide gel electrophoresis (μg per lane), transferred to Hybond ECL nitrocellulose membranes (GE Healthcare), probed with the indicated antibodies and analysed by enhanced chemiluminescence (GE Healthcare). Glyceraldehyde 3-phosphate dehydrogenase and histone H1 were used, respectively, to authenticate the purity of cytoplasmic and nuclear lysates. For a full view of the blots including the location of molecular weight markers, see Supplementary Fig. 3.

### Immunocytochemistry

Cultured cells were fixed in 4% paraformaldehyde in PBS for 15 min then treated for 1 h with blocking buffer containing 4% fetal bovine serum albumin (Sigma-Aldrich) and 0.2% Triton X-100 (VWR) in PBS. Cells were incubated with primary antibodies overnight at 4 °C in blocking buffer, washed (3 × 5 min), and incubated with species-specific secondary antibodies for 1 h at room temperature. Species-specific isotype controls were used for all primary antibodies. Cells were counterstained with 4 μg ml^−1^ Hoechst 33342 or 4 μg ml^−1^ 4′, 6-diamidino-2-phenylindole (DAPI) for 2 min to visualize nuclei. Image acquisition was performed on an Axio Observer Z1 inverted epifluoresence microscope at 20 × magnification using AxioVision v 4.8 (Zeiss).

### Immunohistochemistry

For tissue staining, 10 μm sections were blocked with normal serum (Vector) for 30 min before incubation with antibodies. For the 12-week-time-point hearts, which were paraffin embedded, sections were deparaffinized in xylene, rehydrated through ethanol and antigens were retrieved with citrate buffer (pH 6.0, 1.92 mg ml^−1^ citric acid) in a microwave. Primary antibodies were incubated for 1 h at room temperature or overnight at 4 °C and secondary antibodies for 1 h at room temperature. Sections were mounted in Prolong Gold with DAPI (Invitrogen). Routine imaging and quantification were performed on a Zeiss Axio Observer Z1 widefield fluorescent microscope and confocal imaging with a Leica SP5 or Zeiss LSM-780 microscope. For each combination of donor cell and antibody tested, 200 mOrange-positive donor cells were counted, encompassing at least five fields at 400 × magnification, and the expression of cardiomyocyte, endothelial and SM markers was scored manually by two independent observers blinded to the conditions. Two-way analysis of variance with Bonferonni *post-hoc* tests was used for statistical analysis, with *P*<0.05 as the threshold for significant results.

For details and concentrations of the antibodies used, see Supplementary Table 1.

### Cardiac injury and cell grafting

Cells were transduced in the presence of hexadimethrine bromide (Polybrene, Sigma-Aldrich) with a modified version of the lentiviral vector pLL3.7 (ref. 61), replacing the CMV promoter with the phosphoglycerate kinase promoter and the green fluorescent protein reporter with mOrange. Successfully transduced cells were identified and fractionated by preparative flow sorting. For all four clones investigated, the resulting purity of mOrange^+^ cells was >97%.

For coronary artery ligation^62^,^63^, C57BL/six females aged 10–14 weeks were anesthetized with 2% isofluorane and given buprenorphine (0.05 mg kg^−1^) for analgesia. The trachea was intubated for mechanical ventilation (120 strokes per min, 200 μl stroke volume, Harvard Apparatus). The thorax was opened at the left fourth intercostal space and the proximal left coronary artery was ligated with 8-0 Ethilon suture (Ethicon). Sham-operated animals were treated similarly but did not have the ligature tied. Immediately after infarction, mice received two intramural injections into the infarct border zone using a 30 gauge Hamilton syringe, each with 250,000 mOrange-labelled cells in 10 μl saline (Hamelyn Pharmaceuticals). Control mice were injected with saline alone. The chest was closed with 5-0 Mersilk suture (Ethicon).

For histological analysis, mice were euthanized by cervical dislocation 1, 14 days and after surgery and perfusion-fixed with PBS followed by 4% paraformaldehyde in PBS. The hearts were then removed, bisected longitudinally, fixed in 4% paraformaldehyde for 6 h at 4 °C, then placed in 10% sucrose in distilled water overnight. Hearts were embedded in optimal cutting temperature compound (Sakura) and frozen in liquid nitrogen-cooled isopentane. For immunostaining, 10 μm frozen sections were prepared using a Leica cryostat (Leica Microsystems). For the 12-week time point, hearts were paraffin embedded to improve tissue preservation.

### Re-isolation of donor-derived cells

To isolate donor-derived cardiomyocytes (Fig. 4c), grafted hearts first were subjected to retrograde Langendorff perfusion for 5 min with high-calcium Krebs solution pre-oxygenated with 95% O_2_, 5% CO_2_ containing (mM) 119 NaCl, 4.7 KCl, 0.94 MgSO_4_, 1.2 KH_2_PO_4_, 25 sodium bicarbonate, 11.5 glucose, 5 nitrilotriacetic acid, 1 Ca^2+^, pH 6.95 (ref. 64). Subsequently, 5 min of perfusion was performed with a low-calcium solution containing 50 μM Ca^2+^ and 10 μM 2, 3-butanedione monoxime (BDM, Sigma), pH 6.95. Next, the hearts were subjected to enzymatic digestion by recirculation perfusion for 2 min with low-calcium Krebs solution supplemented with Protease type XXIV (0.182 mg ml^−1^, Sigma), 10 mM BDM, pH 7.4, followed by perfusion with low-calcium solution containing 1 mg ml^−1^ collagenase (Worthington), 0.6 mg ml^−1^ hyaluronidase (Sigma), 10 mM BDM, pH 7.4 (ref. 64). Tissue was then minced and agitated under 100% O_2_ in collagenase for 5–15 min, until digestion of the ventricles was complete. The cell suspension was then centrifuged at low speed and the pellet suspended in low-Ca^2+^ Krebs solution^64^.

A modified protocol was used to analyse α-sarcomeric actin expression in donor-derived cardiomyocytes by immunofluorescence and better preserve their sarcomeric structure (Fig. 4d). Retrograde perfusion was performed in Ca^2+^-free buffer, a recombinant enzyme mix was used (Liberase; Roche), Ca^2+^ re-introduction was performed at room temperature and cells were plated on dishes coated with 2 mg ml^−1^ laminin (Invitrogen).

### Fate mapping

A Cre-lox system was used for fate mapping (see Supplementary Table 3 for the lines used). Typically, F1 double transgenic mice were generated by crossing Cre deleter males with *R26R-tdTom* females, to prevent ectopic reporter expression caused by nonspecific deletion of floxed alleles^65^. Due to the reported ‘parent-of-origin' effect^66^, female *EIIa-Cre* mice were crossed with *R26R-tdTom* males. Because inconsistent recombination even between littermates was reported for several of the Cre strains used (*Tek-Cre, Vav-Cre*^67^), multiple bigenic specimens from at least two independent litters were analysed for all crosses, to avoid possible misinterpretation caused by sample variation, and samples with anomalous reporter activity were excluded from further analysis.

### Magnetic resonance imaging

Mice were imaged using a 9.4 T MRI system (Agilent, Palo Alto, CA, USA) and a 38 mm quadrature-driven birdcage RF coil (Rapid Biomedical, Rimpar, Germany). Multi-slice cardiac and respiratory gated cine-MRI was performed in the true short-axis orientation and covered the whole LV^68^. Late gadolinium-enhanced MRI was performed 20 min after intraperitoneal (i.p.) injection of 0.5 mmol kg^−1^ Gd-DTPA-BMA (Omniscan, GE Healthcare, Hatfield, UK), using a multi-slice inversion recovery sequence^69^. Data were analysed in a blinded fashion using ImageJ (NIH, Bethesda, MD, US). Standard measures of left and right ventricular morphology and function were made from cine stacks, and infarct area determined by thresholding to three s.d. above the mean remote myocardial signal intensity^70^. Infarct size was quantified as epicardial circumferential length of the enhanced tissue, expressed as a percentage of total epicardial circumference of the left ventricle.

### Statistics

The statistical approaches to RNA profiling are given in the sections on qRT–PCR above. For other experimental procedures, unless otherwise stated, Student's two-tailed *t*-test was used for pairwise comparisons and one-way (Fig. 6) or two-way analysis of variance with Bonferroni's correction for multiple comparisons.

## Additional information

**How to cite this article:** Noseda, M *et al.* PDGFRα demarcates the cardiogenic clonogenic Sca1+ stem/progenitor cell in adult murine myocardium. *Nat. Commun.* 6:6930 doi: 10.1038/ncomms7930 (2015).

## Supplementary Material

Supplementary Information Supplementary Figures 1-9, Supplementary Tables 1-3 and Supplementary References
