# Supplementary material for: PDGFRα demarcates the cardiogenic clonogenic Sca1+ stem/progenitor cell in adult murine myocardium
Source: Nat Commun. 2015 May 18;6:6930. doi: 10.1038/ncomms7930 (PMC4479024; doi:10.1038/ncomms7930)
Supplement: Supplementary Information — Supplementary Figures 1-9, Supplementary Tables 1-3 and Supplementary References [file ncomms7930-s1.pdf]

SUPPLEMENTARY INFORMATION

Supplementary Figures:

Supplementary Figure 1

Supplementary Figure 2

Supplementary Figure 3

Supplementary Figure 4

Supplementary Figure 5

Supplementary Figure 6

Supplementary Figure 7

Supplementary Figure 8

Supplementary Figure 9

Supplementary Tables:

Supplementary Table 1

Supplementary Table 2

Supplementary Table 3

Supplementary References

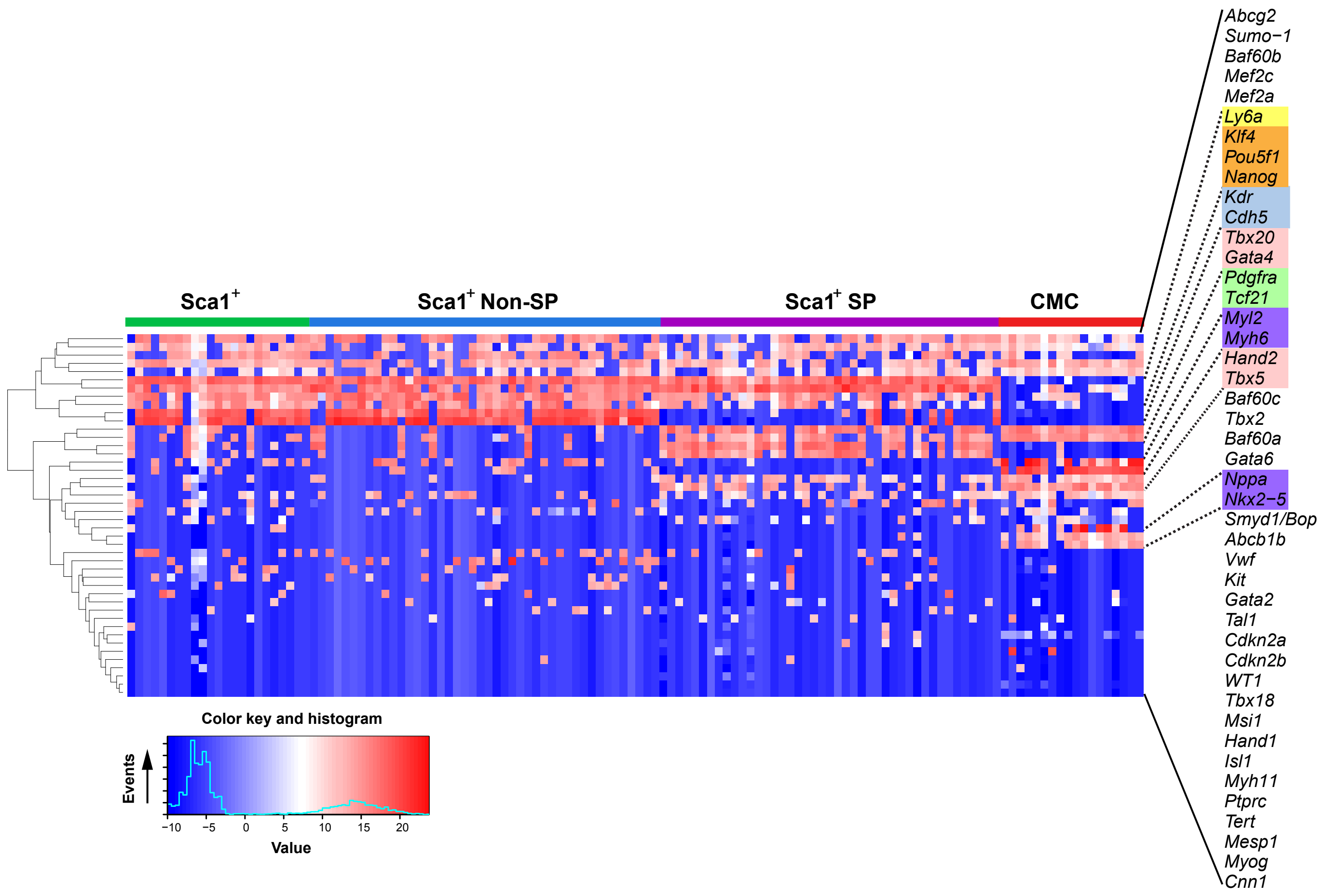

Supplementary Figure 1. Single-cell gene expression profiles of cardiac Sca1 $^{+}$, Non-SP and SP cells, in comparison to cardiomyocytes. Expression of 44 genes was analysed at the single cell level in the four cell populations shown: adult heart-derived Lin^{-} Sca1^{+} cells (*n*=23)**,** Lin^{-} Sca1^{+} Non-SP cells (*n*=44), Lin^{-} Sca1^{+} SP cells (*n*=43) and neonatal cardiomyocytes (CMC, *n*=18)**.** The heat map illustrates expression as -ΔCT values (blue, low or absent; red, high) and hierarchical clustering reveals the similarities of co-expression among functionally related genes. Highlighted genes: *Ly6a* (Sca1), **yellow**; pluripotency genes, **orange**; Cdh5 and Kdr, enriched in Non-SP, **light blue**; *Pdgfra* and *Tcf21,* enriched in SP, **green**; cardiac transcription factors enriched in SP and CMC, **light red**; cardiomyocyte markers, **violet**.

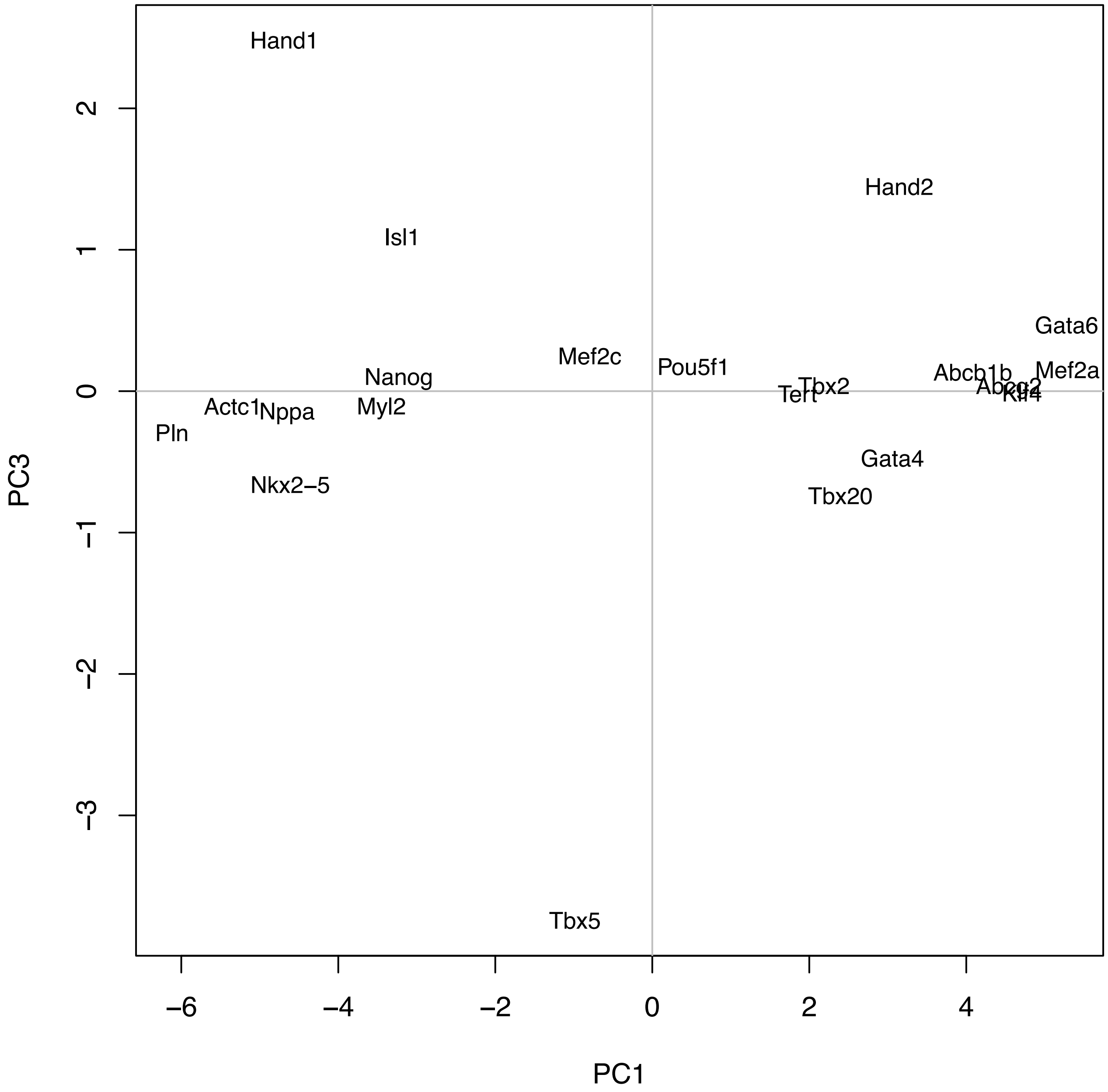

Supplementary Figure 2. Partial representation of the PCA results in Fig. 1d. PC1 accounted for 39% of the variability in the data, but not the constrasting expression profiles of SP versus Non-SP cells.

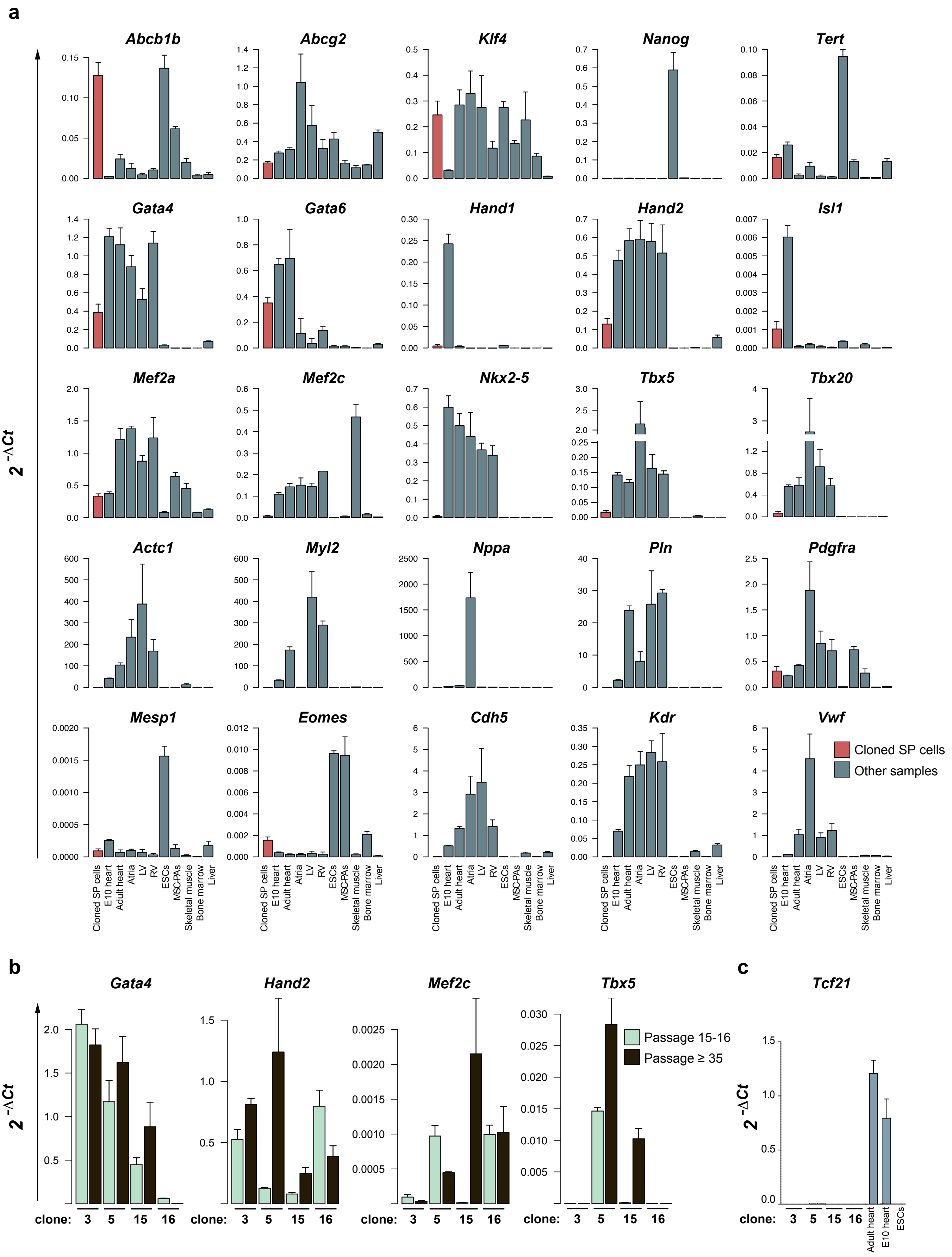

a
Abcb1b
Abcg2
Klf4
Nanog
Tert
Gata4
Gata6
Hand1
Hand2
Isl1
Mef2a
Mef2c
Nkx2-5
Tbx5
Tbx20
Actc1
Myl2
Nppa
Pln
Pdgfra
Mesp1
Eomes
Cdh5
Kdr
Vwf
2 -ΔCt
Cloned SP cells
Other samples
Cloned SP cells
E10 heart
Adult heart
Atria
LV
RV
ESCs
MSCPAs
Skeletal muscle
Bone marrow
Liver
b
Gata4
Hand2
Mef2c
Tbx5
Passage 15-16
Passage ≥ 35
clone: 3 5 15 16
c
Tcf21
Adult heart
E10 heart
ESCs

Supplementary Figure 3. qRT-PCR profiling of cardiac SP clones. (**a**) Expression profiles using custom TaqMan low density arrays (TLDA) are shown for the indicated genes in 20 independent cardiac SP clones (red) and reference samples (grey). Stem cell-associated: *Abcb1*, *Abcg2*, *Klf4*, *Nanog*, *Tert*. Cardiogenic transcription factors: *Gata4, Gata6, Hand1, Hand2, Isl1, Mef2a, Mef2c, Nkx2-5, Tbx5, Tbx20*. Cardiomyocyte: *Actc1*, *Myl2*, *Nppa*, *Pln*. Mesoderm: *Eomes*, *Mesp1*, *Pdgfra*. Vascular: *Cdh5*, *Kdr, Vwf*. Cardiogenic transcription factor expression was significantly enriched in cardiac SP clones, relative to MSCs, as follows: *Gata4*, $p \leq 0.01$; *Mef2a*, *Nkx2-5*, *Tbx5*, $p \leq 0.005$; *Gata6*, *Hand2*, *Isl1*, $p \leq 0.001$. (**b**) Expression of selected genes by TLDA is compared in clones harvested at passage 15-16 (green) and after passage 35 (black). (**c**) *Tcf21* expression in the samples used for panel **b** was determined by standard qRT-PCR in multiplex with *Hmbs* as a loading control. Abbreviations: LV, left ventricle; RV, right ventricle; MSCs, PDGFRα^+ bone marrow MSCs. Student's two-tailed t-test was used. Error bars indicate the SEM between independent experiments. $n \geq 3$.

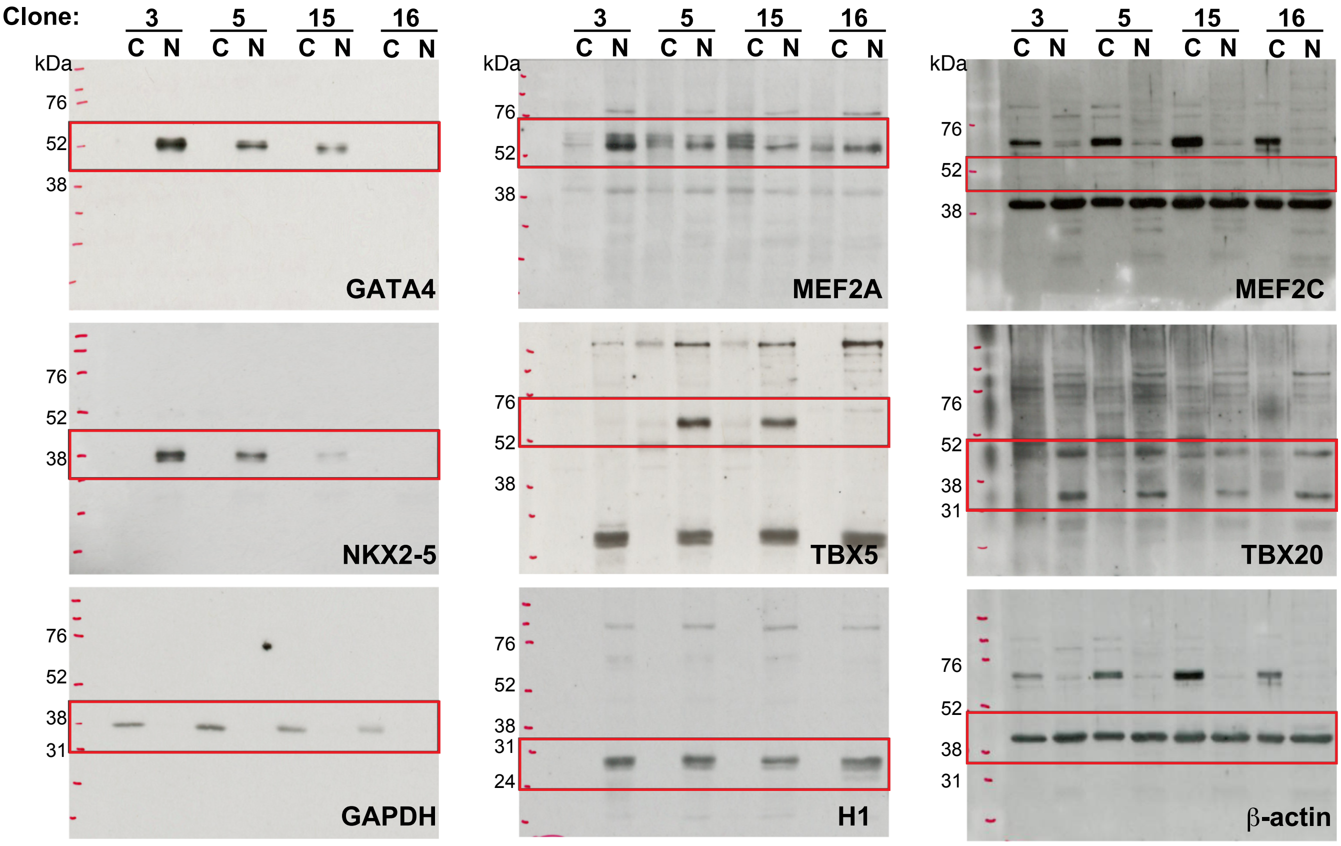

Supplementary Figure 4. Full view of the Western blots shown in Figure 3e of the main manuscript. The uncropped blots are accompanied by the location of the molecular weight markers. The indicated cardiac transcription factors and control proteins were compared in the Cytoplasmic (C) versus Nuclear (N) fractions of four cloned CSC lines, as shown. GAPDH and histone H1 authenticate the C and N fractions, respectively, and β-actin corroborates equivalent loading of each sample. Boxes (red) indicate the cropped areas shown in Fig. 3e.

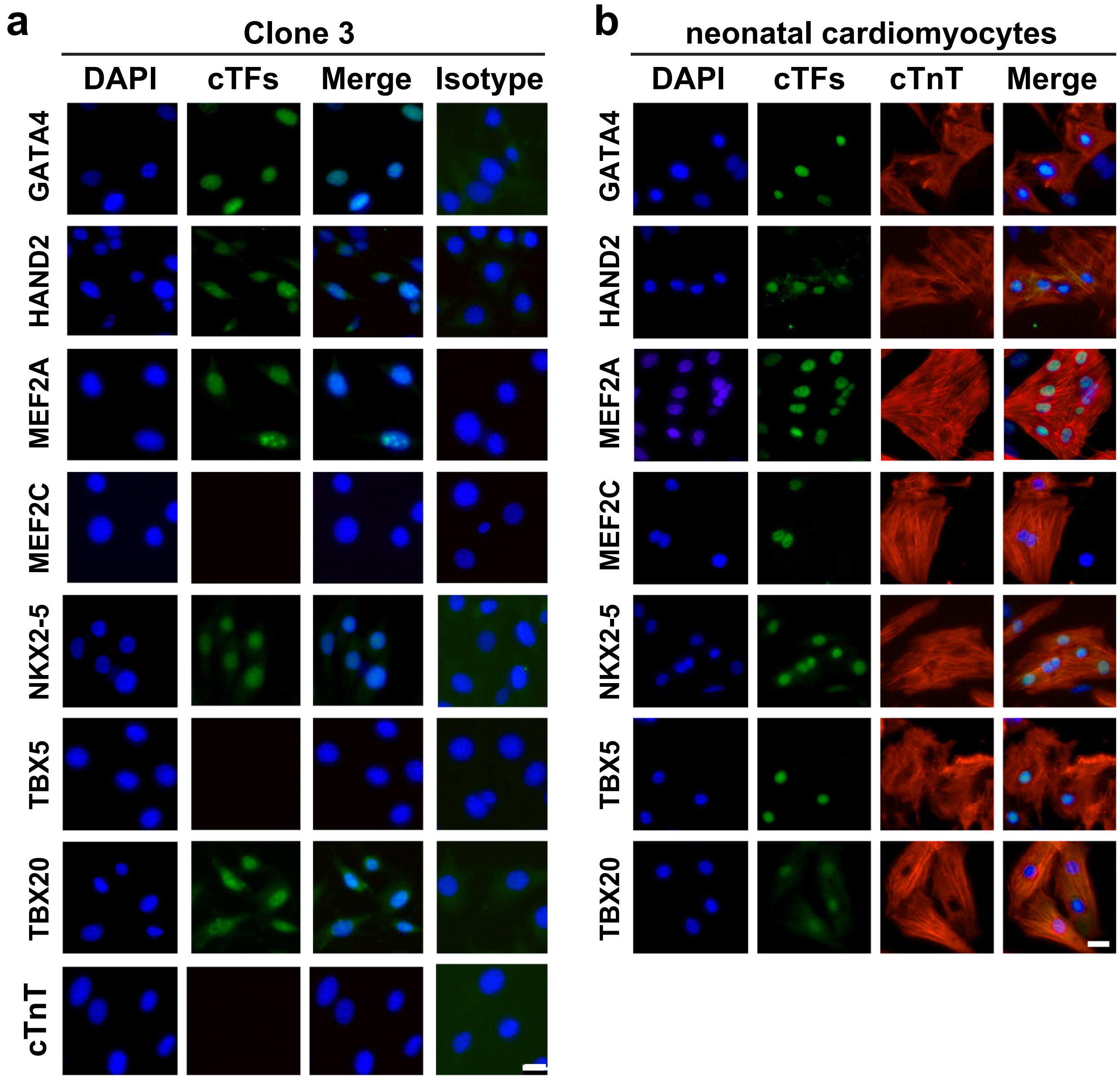

Supplementary Figure 5. Immunolocalization of cardiogenic transcription factors in cloned cardiac SP cells. Immunofluorescence microscopy of cloned cardiac SP cells (**a**) and neonatal cardiomyocytes (**b**). Green, cardiac transcription factors (cTFs) imaged with Alexa488-conjugated secondary antibodies; red, cardiac troponin T (cTnT) imaged with Alexa 647-conjugated secondary antibody; blue, DAPI. All transcrIption factors expressed at the RNA level were present as nuclear-localized proteins. Isotype controls for all antibodies used are shown in panel **a**, column 4, and the absence of cTnT in SP cells in panel **a**, row 8. Bar, 20 μm.

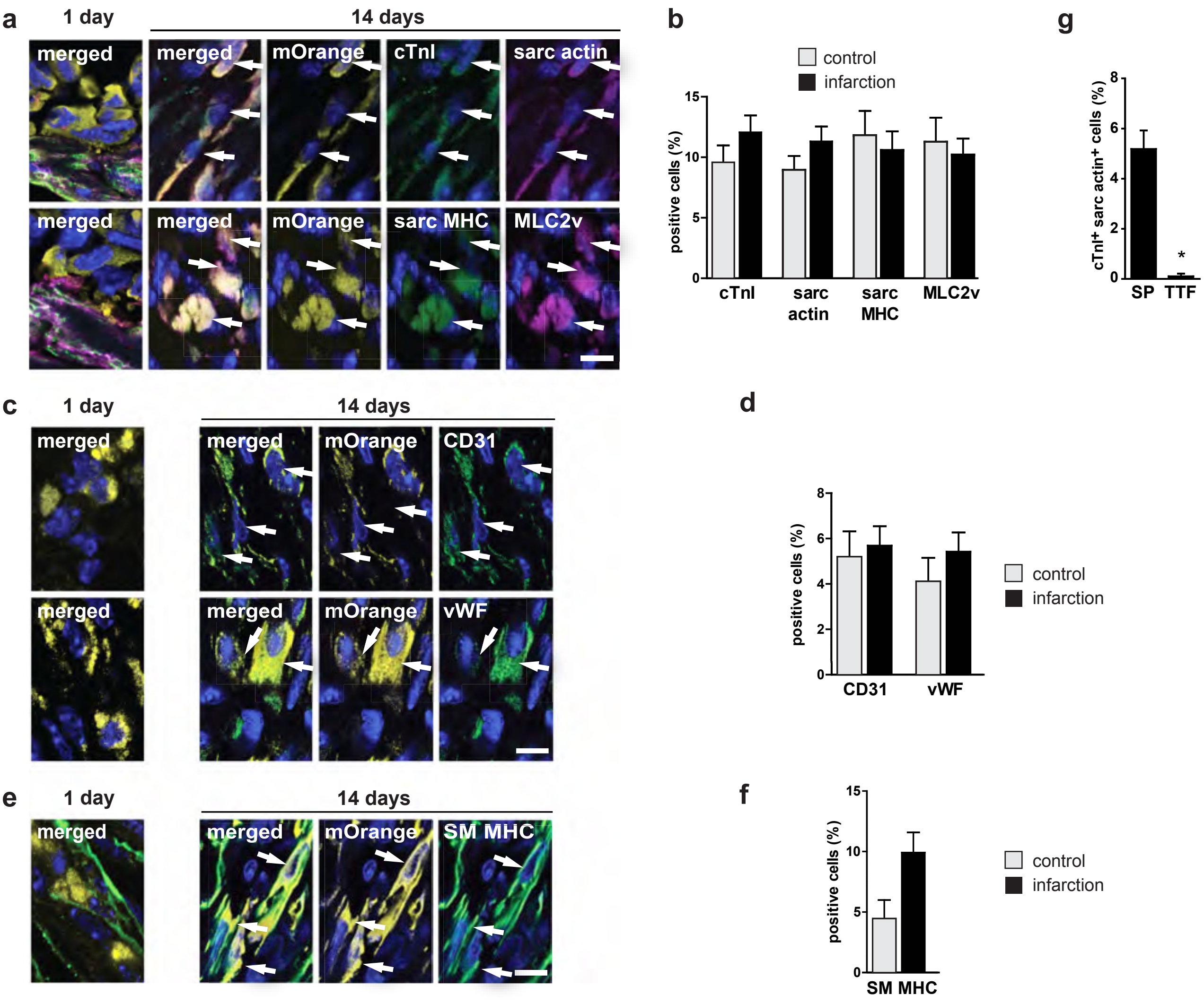

Supplementary Figure 6. Induction of cardiac, smooth muscle, and endothelial markers in single-cell derivatives of adult cardiac SP cells after grafting. Four independent clones (3, 5, 15 and 16) were transduced with mOrange and delivered by intramural injection in sham-operated mice (control) or infarcted mice at the time of coronary artery ligation (infarction). Images are for clone 3 (**a**, **c**, **e**). For samples 1 day after injection, only merged images are shown. Arrowheads highlight donor cells expressing the cardiovascular markers tested. Bar, 20 μm. Similar results were obtained with all four clonal lines (**b**, **d**, **f**). At least 200 cells were scored for each clone and each condition shown (≥ 800 cells per marker), using 3 sections ≥ 80 μm apart and containing mOrange^{+} cells. *n* = 3-4 for sham-operated hearts and 5-7 for infarcted ones. (**a**, **b**) Induction of cardiac troponin I (cTnI, green) and sarcomeric α-actin (sarc actin, violet; above) or sarcomeric myosin heavy chains (sarc MHC) and myosin light chain 2v (MLC2v; below) in grafted cells at 14 days. (**c**, **d**) Induction of CD31 and von Willebrand factor (vWF) factor (green). (**e**, **f**) Induction of smooth muscle myosin heavy chain (SM MHC, green). Error bars indicate the SEM between independent experiments and combine results for all four clones. (**g**) Mouse tail tip fibroblats (TTF), lentivirally transduced to express mOrange, were injected at the time of coronary artery ligation to test their capacity to express cardiac markers as comparison to adult cardiac SP cells (SP). Results are the percentage of mOrange^{+} cells co-expressing the two structural markers, cTnI and sarc actin, 2 weeks after injection. Bars represent the mean of 26 independent injections for cardiac SP cells (clones 3, 5,15 and 16) and 5 for fibroblasts. *, $p \leq 0.01$ by Student's two tailed t-test.

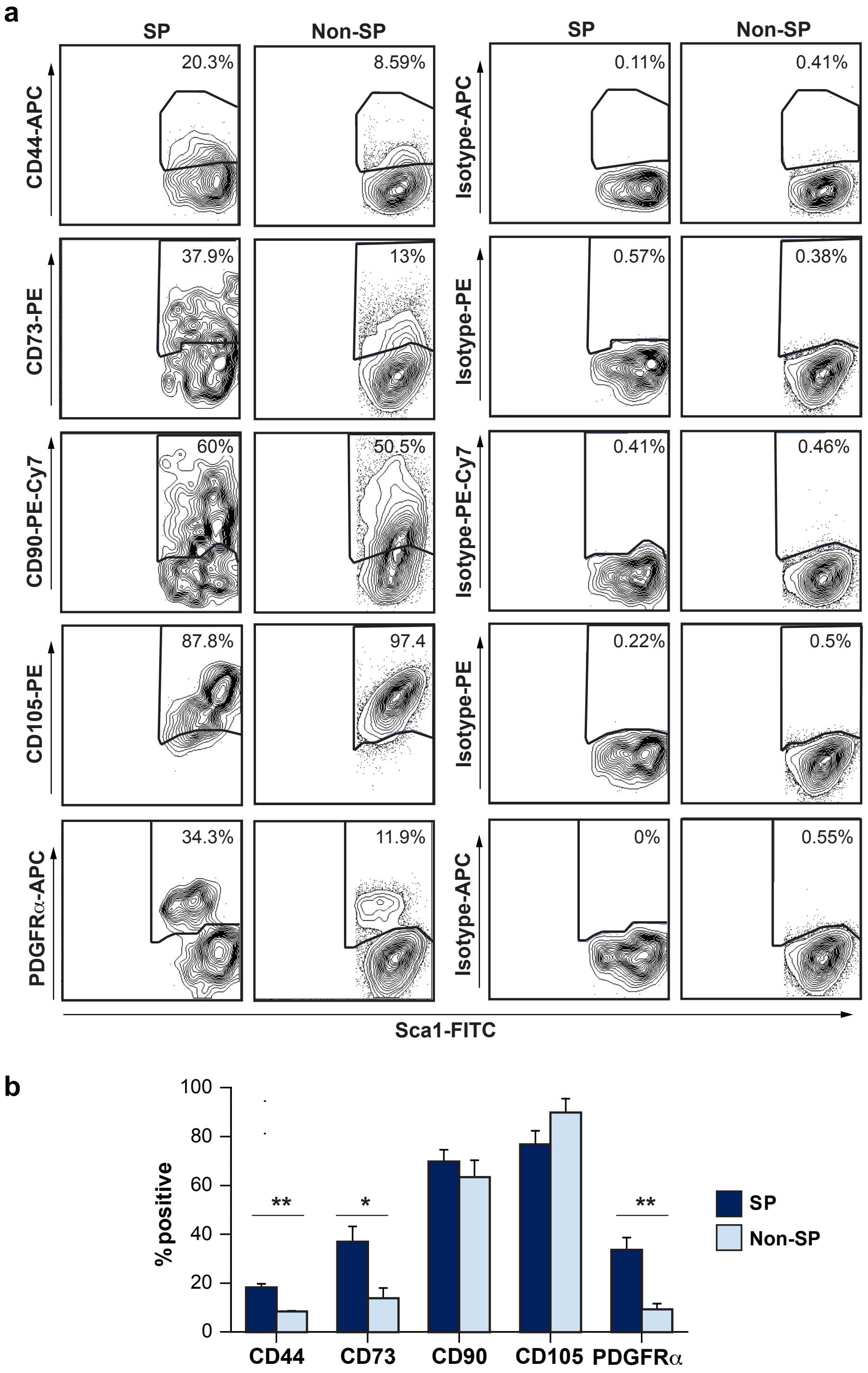

Supplementary Figure 7. The MSC-like immunophenotype of Sca1$^+$ SP vs Non-SP cells. Freshly isolated Lin$^-$/Sca1$^+$ cardiac cells were stained with Hoechst 33342 for SP status and with conjugated antibodies against the indicated surface proteins (CD44, CD73, CD90, CD105, PDGFRα), then were analyzed by flow cytometry. (**a**) Left, Immunostaining is shown as contour plots for the SP and non-SP fractions. Right, Negative results are shown for the corresponding conjugated isotype controls. (**b**) Mean proportion of cardiac SP and non-SP cells expressing the indicated markers. Error bars indicate the SEM between independent experiements. n = 3. *, $p \le 0.05$; **, $p \le 0.01$ by Student's two-tailed t-test. Abbreviations: APC, allophycocyanin; Cy, cyanine; PE, phycoerythrin.

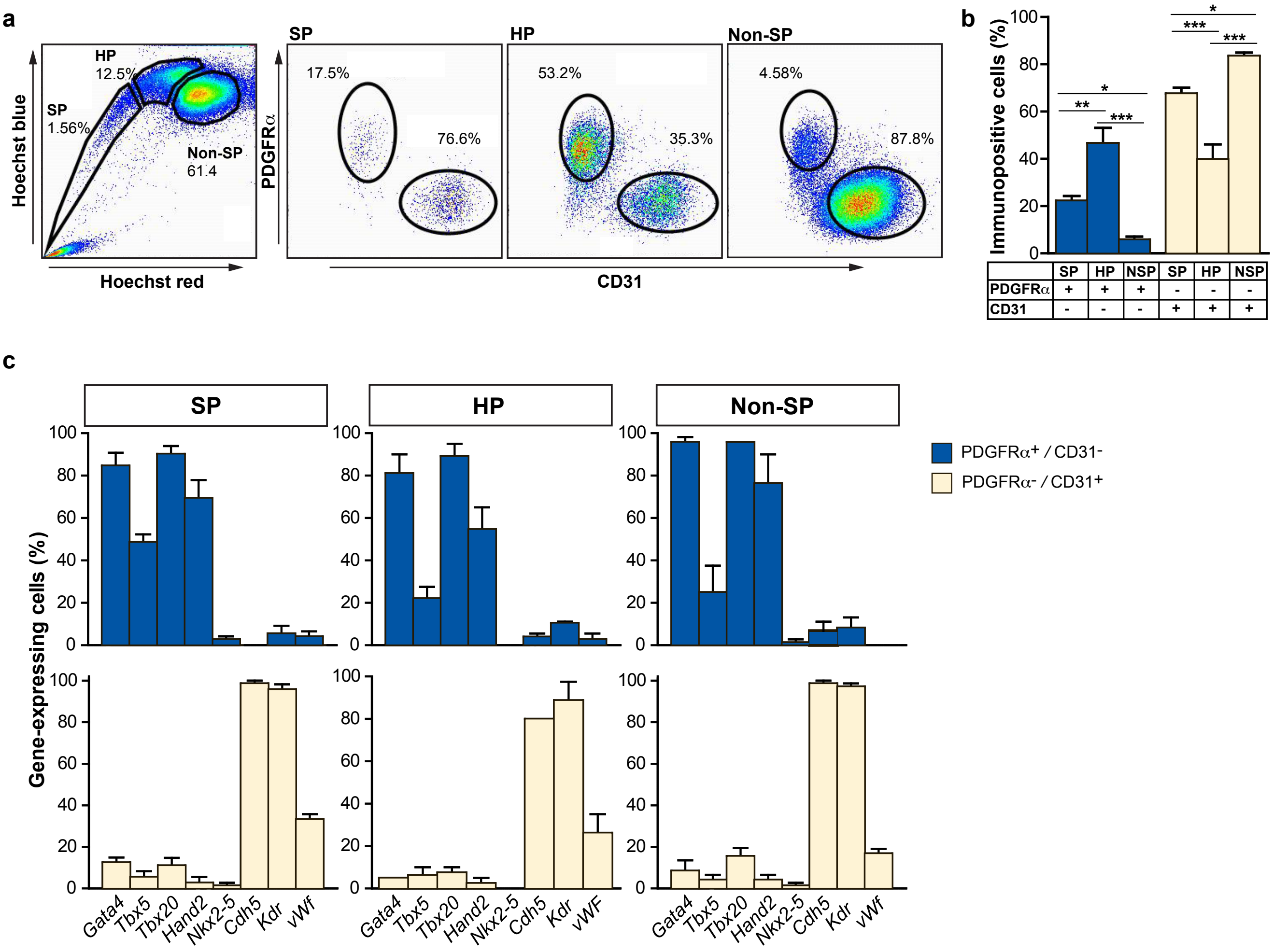

Supplementary Figure 8. PDGFRα and the cardiogenic signature in the "hinge" population. Freshly isolated Lin$^-$/Sca1$^+$ cardiac cells were stained with Hoechst 33342 for the SP phenotype and with conjugated antibodies against CD31 and PDGFRα, then single cells were sorted by flow cytometry for single cell qRT- PCR. (**a**) Density plots illustrating the gating strategy for SP, hinge population (HP) and Non-SP cells and the six respective subpopulations partitioned by PDGFRα and CD31. Data shown here are from the experiment in Fig. 6, analysed with an adjusted gating strategy to include the intermediate hinge population. (**b**) Mean proportion of cardiac SP, HP and Non-SP cells expressing the indicated markers. Error bars indicate the SEM from 6 independent experiments. One way ANOVA with the Tukey posthoc test was used for the intergroup comparisons. *, $p \le 0.05$; **, $p \le 0.01$; ***$p \le 0.001$. (**c**) Frequency of expression of the indicated genes in the PDGFRα$^+$/CD31$^-$ (blue) versus PDGFRα$^-$/CD31$^+$ (yellow) subpopulations of cardiac SP, HP and non-SP cells.

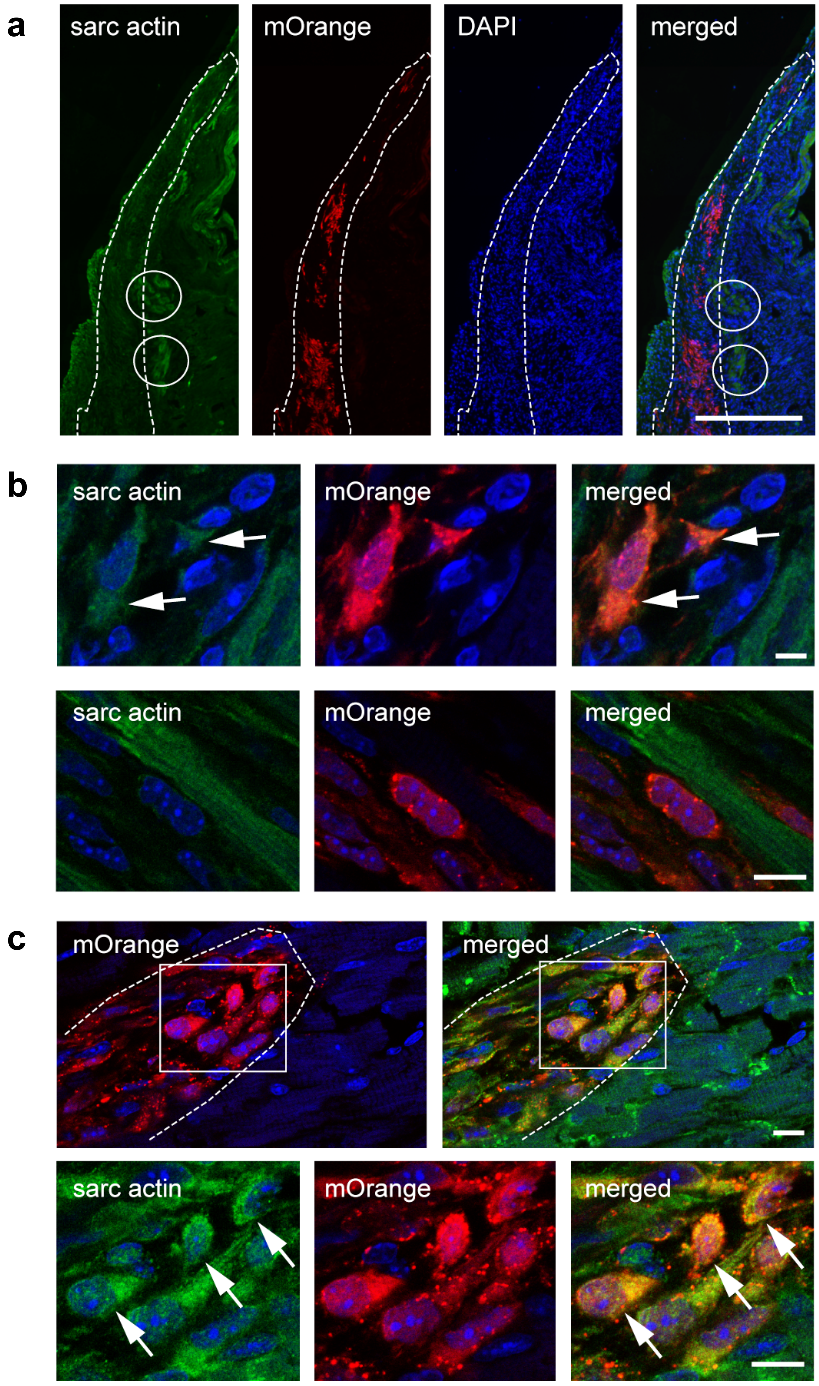

Supplementary Figure 9. The presence of PDGFRα plus lack of CD31 identifies Sca1$^+$ cells with in vivo cardiogenic potential. Uncloned Lin$^-$/Sca1$^+$/PDGFRα^+/CD31$^-$ cells were expanded for ≤10 passages. Hearts were analyzed 2 weeks after injection by confocal microscopy for mOrange (red) and α-sarcomeric actin (green). Nuclei were stained with DAPI (blue). (**a**, **b**) Cells were injected at the time of coronary artery ligation. (**a**) Location of the graft (dotted line) relative to the infarcted area. Islands of surviving myocytes are highlighted (circles). (**b**) Above, co-localization of α-sarcomeric actin and mOrange in two donor-derived cells (arrows). Below, a sarcomeric actin-negative donor-derived cell from the same section. (**c**) Cells were injected at the time of sham operation. Above, a region of the mOrange$^+$ graft within the uninjured myocardium. Below, the highlighted portion of this graft is shown at higher magnification, with co-localization of α-sarcomeric actin and mOrange seen in three cells (arrows). Bar: a, 500 μm; b, c, 10 μm.

Supplementary Table 1. List of antibodies

PRIMARY ANTIBODIES

Antigen	Species raised	Conjugate	Application (concentration)	Company	Product code	Country
CD31	Rat	FITC	FACS (2.5 $\mu g \cdot ml^{-1}$)	BD Biosciences	553372	UK
CD44	Rat	APC	FACS (0.5-2 $\mu g \cdot ml^{-1}$)	BD Biosciences	559250	UK
CD73	Rat	PE	FACS (0.5-2 $\mu g \cdot ml^{-1}$)	BD Biosciences	550741	UK
CD90.2	Rat	PE-Cy7	FACS (0.5-2 $\mu g \cdot ml^{-1}$)	eBioscience	25-0902	UK
CD105	Rat	PE	FACS (0.5-2 $\mu g \cdot ml^{-1}$)	BD Biosciences	562759	UK
CD140a	Rat	APC	FACS (4 $\mu g \cdot ml^{-1}$)	BioLegend	135908	UK
Lineage cocktail	Rat	Biotin	AutoMACS (10 μl)	Miltenyi	130-090-858	UK
Sca1	Rat	FITC	AutoMACS (10 μl)	Miltenyi	130-092-529	UK
Sca1	Rat	APC	FACS (0.5-1 $\mu g \cdot ml^{-1}$)	BioLegend	108112	UK
Sca1	Rat	PE	FACS (0.5-1 $\mu g \cdot ml^{-1}$)	BioLegend	108108	UK
BAF60c	Rabbit	--	WB (0.6 $\mu g \cdot ml^{-1}$), IF (2.9 $\mu g \cdot ml^{-1}$)	ProtTech	12838-1-AP	US
β-actin	Rabbit	--	WB (0.008 $\mu g \cdot ml^{-1}$)	Cell Signaling / New England Biolabs	4967	UK
GATA4	Mouse	--	WB (1 $\mu g \cdot ml^{-1}$), IF (10 $\mu g \cdot ml^{-1}$)	R&D	MAB2606	US
GAPDH	Rabbit	--	WB (0.0242 $\mu g \cdot ml^{-1}$)	Cell Signaling / New England Biolabs	2118	UK
Histone H1	Mouse	--	WB (7.50 $\mu g \cdot ml^{-1}$)	Millipore	MABE71	UK
HAND2	Goat	--	WB (1 $\mu g \cdot ml^{-1}$), IF (2 $\mu g \cdot ml^{-1}$)	R&D	AF3876	US
HSP90	Rabbit	--	WB (0.014 $\mu g \cdot ml^{-1}$)	Cell Signaling / New England Biolabs	4877	UK
MEF2A	Rabbit	--	WB (1-3 $\mu g \cdot ml^{-1}$), IF (5-15 $\mu g \cdot ml^{-1}$)	Abcam	ab76063	UK
MEF2C	Rabbit	--	WB (0.2 $\mu g \cdot ml^{-1}$), IF (0.84)	Cell Signaling / New England Biolabs	5030	UK
NKX2-5	Goat	--	WB (1 $\mu g \cdot ml^{-1}$), IF (1 $\mu g \cdot ml^{-1}$)	Santa Cruz	sc8697	DE
TBX5	Mouse	--	WB (4 $\mu g \cdot ml^{-1}$), IF (2.5 $\mu g \cdot ml^{-1}$)	Abnova	HRP0006910-M01	DE
TBX20	Rabbit	--	WB (0.5 $\mu g \cdot ml^{-1}$), IF (1 $\mu g \cdot ml^{-1}$)	Orbigen	ARP-PAB11248	US
CD31	Rat	FITC	IF (0.5 $\mu g \cdot ml^{-1}$)	BD Bioscience	553372	UK
cTnI	Goat	--	IF (4 $\mu g \cdot ml^{-1}$)	Santa Cruz	sc8118	DE

cTnT	Mouse	--	IF (1 $\mu g \cdot ml^{-1}$)	Santa Cruz	sc52284	DE
ds Red	Rabbit	--	IF (0.5 $\mu g \cdot ml^{-1}$)	Clontech	632496	US
MLC2v	Mouse	Alexa Fluor 647	IF (2 $\mu g \cdot ml^{-1}$)	Synaptic Systems	310-111AT1	DE
SM-MyHC	Rabbit	Alexa Fluor 488	IF (1.25 $\mu g \cdot ml^{-1}$)	Biomedical Technologies	BT-562	US
Sarc MyHC	Mouse	FITC	IF (1.25 $\mu g \cdot ml^{-1}$)	R&D	MAB4470	US
Sarc actin	Mouse	--	IF (6-12 $\mu g \cdot ml^{-1}$)	Sigma	A2172	UK
vWF	Rabbit	Alexa Fluor 488	IF (2 $\mu g \cdot ml^{-1}$)	Dako	A008229	UK

SECONDARY ANTIBODIES AND ASSOCIATED REAGENTS

Antigen	Species raised	Conjugate	Application (concentration)	Company	Product code	Country
Goat IgG	Rabbit	HRP	WB (0.2 $\mu g \cdot ml^{-1}$)	Abcam	ab5755	UK
Mouse IgG	Horse	HRP	WB (0.05-0.17 $\mu g \cdot ml^{-1}$)	Cell Signaling / New England Biolabs	7076	UK
Rabbit IgG	Goat	HRP	WB (0.1 $\mu g \cdot ml^{-1}$)	Abcam	ab6112	UK
Goat IgG	Chicken	Alexa Fluor 488	IF (2 $\mu g \cdot ml^{-1}$)	Molecular Probes	A21467	UK
Goat IgG	Donkey	Alexa Fluor 555	IF (2 $\mu g \cdot ml^{-1}$)	Molecular Probes / Life Technologies	A11057	US
Goat IgG	Donkey	Alexa Fluor 647	IF (3 $\mu g \cdot ml^{-1}$)	Jackson Immunolabs / Stratech	705-606-147	UK
Goat IgG	Rabbit	Alexa Fluor 488	IF (5 $\mu g \cdot ml^{-1}$)	Invitrogen / Life Technologies	A11078	US
Mouse IgG	Donkey	Alexa Fluor 647	IF (3 $\mu g \cdot ml^{-1}$)	Jackson Immunolabs / Stratech	715-495-150	UK
Mouse IgG	Goat	Alexa Fluor 555	IF (2 $\mu g \cdot ml^{-1}$)	Cell Signaling / New England Biolabs	4409	UK
Mouse IgG	Goat	Alexa Fluor 488	IF (2 $\mu g \cdot ml^{-1}$)	Cell Signaling / New England Biolabs	4408	UK
Mouse IgM	Goat	Alexa Fluor 647	IF (5 $\mu g \cdot ml^{-1}$)	Invitrogen / Life Technologies	A21238	US
Rabbit IgG	Donkey	Alexa Fluor 647	IF (3 $\mu g \cdot ml^{-1}$)	Jackson Immunolabs / Stratech	711-606-152	UK
Rabbit IgG	Goat	Alexa Fluor 488	IF (2 $\mu g \cdot ml^{-1}$)	Cell Signaling / New England Biolabs	4412	UK
Rabbit IgG	Goat	Alexa Fluor 594	IF (5 $\mu g \cdot ml^{-1}$)	Invitrogen / Life Technologies	A21244	US

Rabbit IgG	Goat	Alexa Fluor 555	IF (2 $\mu g \cdot ml^{-1}$)	Cell Signaling / New England Biolabs	4413	UK
SA	--	APC	FACS (1 $\mu g \cdot ml^{-1}$)	eBioscience	17-4317	UK
SA	--	PE-Cy7	FACS (0.5-1 $\mu g \cdot ml^{-1}$)	eBioscience	25-4317	UK

Abbreviations: APC, allophycocyanin; cTn, cardiac troponin; Cy, cyanine; FACS, fluorescence-activated cell sorting; FITC, fluorescein isothiocyanate; HRP, horseradish peroxidase; IF, immunofluorescence microscopy; MyHC, myosin heavy chain; PE, phycoerythrin; SA, streptatidin; sarc, sarcomeric; SM, smooth muscle; WB, Western blotting. The lineage depletion cocktail comprises biotinylated antibodies to CD5, CD45R (B220), CD11b, Anti-Gr-1 (Ly-6G/C), 7-4, and Ter-119. Concentrations in parentheses are the working concentration of each antibody ($\mu g \cdot ml^{-1}$, where available, or manufacturer's recommendation in μl in the case of proprietary solutions).

Supplementary Table 2. TaqMan assays

Gene	TaqMan primers/probes	Applications	Gene	TaqMan primers/probes	Applications
Abcb1b	Mm01324120_m1	qRT-PCR; single-cell	*Msi1*	Mm00485224_m1	qRT-PCR; single-cell
Abcg2	Mm00496364_m1	qRT-PCR; single-cell	*Myh11*	Mm00443013_m1	qRT-PCR; single-cell
Actc1	Mm01333821_m1	qRT-PCR	*Myh6*	Mm00440359_m1	qRT-PCR; single-cell
Baf60a	Mm00473402_m1	qRT-PCR; single-cell	*Myl2*	Mm00440384_m1	qRT-PCR; single-cell
Baf60b	Mm00473467_g1	qRT-PCR; single-cell	*Myog*	Mm00446194_m1	qRT-PCR; single-cell
Baf60c	Mm00491850_m1	qRT-PCR; single-cell	*Nanog*	Mm02019550_s1	qRT-PCR; single-cell
Bmp2	Mm01340178_m1	qRT-PCR; single-cell	*Nkx2-5*	Mm00657783_m1	qRT-PCR; single-cell
Bmp4	Mm00432087_m1	qRT-PCR; single-cell	*Nppa*	Mm01255748_g1	qRT-PCR; single-cell
Cdh5	Mm00486938_m1	qRT-PCR; single-cell	*Pdgfra*	Mm01211685_m1	qRT-PCR; single-cell
Cdkn2a	Mm01257348_m1	qRT-PCR; single-cell	*Pln*	Mm00452263_m1	qRT-PCR
Cdkn2b	Mm00483241_m1	qRT-PCR; single-cell	*Pou5f1*	Mm03053917_g1	qRT-PCR; single-cell
Cer1	Mm03024044_m1	qRT-PCR	*Ptprc*	Mm01293577_m1	qRT-PCR; single-cell
Cnn1	Mm00487032_m1	qRT-PCR; single-cell	*RN18S1*	Hs99999901_s1	qRT-PCR; single-cell
Eomes	Mm01351986_m1	qRT-PCR	*Ryr2*	Mm00465877_m1	qRT-PCR
Foxa1	Mm00484713_m1	qRT-PCR	*Smyd1/Bop*	Mm00477663_m1	qRT-PCR; single-cell
Gata2	Mm00492300_m1	qRT-PCR; single-cell	*Sox17*	Mm00488363_m1	qRT-PCR
Gata4	Mm00484689_m1	qRT-PCR; single-cell	*Sumo1*	Mm01609844_g1	qRT-PCR; single-cell
Gata6	Mm00802636_m1	qRT-PCR; single-cell	*T*	Mm00436877_m1	qRT-PCR
Hand1	Mm00433931_m1	qRT-PCR; single-cell	*Tal1*	Mm01187033_m1	qRT-PCR; single-cell
Hand2	Mm00439247_m1	qRT-PCR;	*Tbx1*	Mm00448948_m1	qRT-PCR

		single-cell			
Hhex	Mm00433954_m1	qRT-PCR	*Tbx18*	Mm00470177_m1	qRT-PCR; single-cell
Hmbs	Mm01143545_m1	qRT-PCR; single-cell	*Tbx2*	Mm00436915_m1	qRT-PCR; single-cell
Isl1	Mm00627860_m1	qRT-PCR; single-cell	*Tbx20*	Mm00451515_m1	qRT-PCR; single-cell
Kdr	Mm00440111_m1	qRT-PCR; single-cell	*Tbx5*	Mm00803518_m1	qRT-PCR; single-cell
Kit	Mm00445212_m1	qRT-PCR; single-cell	*Tcf21*	Mm00448961_m1	qRT-PCR; single-cell
Klf4	Mm00516104_m1	qRT-PCR; single-cell	*Tert*	Mm01352136_m1	qRT-PCR; single-cell
Ly6a	Mm00726565_s1	qRT-PCR; single-cell	*Tmsb4x*	Mm01161568_m1	qRT-PCR; single-cell
Mef2c	Mm01340842_m1	qRT-PCR; single-cell	*Ubc*	Mm01201237_m1	qRT-PCR; single-cell
Mefa	Mm01318991_m1	qRT-PCR; single-cell	*Vwf*	Mm00550376_m1	qRT-PCR; single-cell
Mesp1	Mm00801883_g1	qRT-PCR; single-cell	*Wt1*	Mm00460570_m1	qRT-PCR; single-cell

Abbreviations: qRT-PCR, quantitative real-time reverse transcriptase-PCR performed manually or in TaqMan low-density array cards; single-cell, single-cell qRT-PCR (Fluidigm).

Supplementary Table 3. Fate-mapping lines

Name	Full name (MGI/JAX)	Model	Principal lineage	Strain	Reference
EIIa-Cre	Tg(EIIa-cre)C5379Lmgd	Tg	Germ line	C57BL/6	1
Flk1-Cre	Kdr$^{tm1(cre)Sato}$/J	KI	Blood and vascular endothelial cells	mixed	2
cGata5-Cre	Tg(GATA5-cre)1Krc	Tg	Epicardial derivatives	C57BL/6	3
Isl1-Cre	Isl1$^{tm1(cre)Sev}$	KI	Second heart field	C57BL/6	4
Mef2c$_{AHF}$-Cre	Tg(Mef2c-cre)2Blk	Tg	Anteriori heart field	mixed	5
Mesp1-Cre	Mesp1$^{tm2(cre)Ysa}$	KI	Mesoderm	C57BL/6	6
Myh6-Cre	Tg(Myh6-cre)2182Mds	Tg	Cardiomyocyte	C57BL/6	7
Nkx2.5-Cre	Nkx2-5$^{tm1(cre)Rjs}$	KI	Cardiac progenitor	C57BL/6	8
R26R-tdTomato	*Gt(ROSA)26Sor$^{tm14(CAG-dTomato)Hze}$*	KI	n/a (Cre-dependent reporter)	C57BL/6	9
Tie2-Cre	Tg(Tek-cre)1Ywa/J	Tg	Vascular endothelial cells	C57BL/6	10
Vav-Cre	Tg(Vav1-cre)1Graf	Tg	Hematopoietic	C57BL/6	11
Wnt1-Cre	Tg(Wnt1-cre)11Rth	Tg	Neural crest	C57BL/6	12
Wt1-CreERT2	Wt1$^{tm2(cre/ERT2)Wtp}$	KI	Epicardium	mixed	13

Abbreviations: JAX, Jackson Laboratory; KI, knock-in; MGI, Mouse Genome Informatics Institute; n/a, not applicable; SHF, second heart field; Tg, transgenic

SUPPLEMENTARY REFERENCES

1. Lakso, M. *et al.* Efficient in vivo manipulation of mouse genomic sequences at the zygote stage. *Proc Natl Acad Sci U S A* **93**, 5860-5865 (1996).
2. Motoike, T., Markham, D.W., Rossant, J. & Sato, T.N. Evidence for novel fate of Flk1+ progenitor: Contribution to muscle lineage. *Genesis* **35**, 153-159 (2003).
3. Zamora, M., Manner, J. & Ruiz-Lozano, P. Epicardium-derived progenitor cells require beta-catenin for coronary artery formation. *Proc Natl Acad Sci U S A* **104**, 18109-18114 (2007).
4. Yang, L. *et al.* Isl1Cre reveals a common Bmp pathway in heart and limb development. *Development* **133**, 1575-1585 (2006).
5. Verzi, M.P., McCulley, D.J., De Val, S., Dodou, E. & Black, B.L. The right ventricle, outflow tract, and ventricular septum comprise a restricted expression domain within the secondary/anterior heart field. *Dev Biol* **287**, 134-145 (2005).
6. Saga, Y. *et al.* MesP1 is expressed in the heart precursor cells and required for the formation of a single heart tube. *Development* **126**, 3437-3447 (1999).
7. Agah, R. *et al.* Gene recombination in postmitotic cells. Targeted expression of Cre recombinase provokes cardiac-restricted, site-specific rearrangement in adult ventricular muscle in vivo. *J Clin Invest* **100**, 169-179 (1997).
8. Moses, K.A., DeMayo, F., Braun, R.M., Reecy, J.L. & Schwartz, R.J. Embryonic expression of an Nkx2-5/Cre gene using ROSA26 reporter mice. *Genesis* **31**, 176-180 (2001).
9. Madisen, L. *et al.* A robust and high-throughput Cre reporting and characterization system for the whole mouse brain. *Nat Neurosci* **13**, 133-140 (2010).
10. Kisanuki, Y.Y. *et al.* Tie2-Cre transgenic mice: a new model for endothelial cell-lineage analysis in vivo. *Dev Biol* **230**, 230-242 (2001).
11. Stadtfeld, M. & Graf, T. Assessing the role of hematopoietic plasticity for endothelial and hepatocyte development by non-invasive lineage tracing. *Development* **132**, 203-213 (2005).
12. Danielian, P.S., Muccino, D., Rowitch, D.H., Michael, S.K. & McMahon, A.P. Modification of gene activity in mouse embryos in utero by a tamoxifen-inducible form of Cre recombinase. *Curr Biol* **8**, 1323-1326 (1998).
13. Zhou, B. *et al.* Epicardial progenitors contribute to the cardiomyocyte lineage in the developing heart. *Nature* **454**, 109-113 (2008).
